# Analyses of binding partners and functional domains for the developmentally essential protein Hmx3a/HMX3

**DOI:** 10.1038/s41598-023-27878-9

**Published:** 2023-01-20

**Authors:** William Haws, Samantha England, Ginny Grieb, Gabriela Susana, Sophie Hernandez, Hunter Mirer, Katharine Lewis

**Affiliations:** Department of Biology, Syracuse University, 107 College Place, Syracuse, NY 13244 USA

**Keywords:** Protein-protein interaction networks, Genetics of the nervous system, High-throughput screening

## Abstract

HMX3 is a homeodomain protein with essential roles in CNS and ear development. Homeodomains are DNA-binding domains and hence homeodomain-containing proteins are usually assumed to be transcription factors. However, intriguingly, our recent data suggest that zebrafish Hmx3a may not require its homeodomain to function, raising the important question of what molecular interactions mediate its effects. To investigate this, we performed a yeast two-hybrid screen and identified 539 potential binding partners of mouse HMX3. Using co-immunoprecipitation, we tested whether a prioritized subset of these interactions are conserved in zebrafish and found that Tle3b, Azin1b, Prmt2, Hmgb1a, and Hmgn3 bind Hmx3a. Next, we tested whether these proteins bind the products of four distinct *hmx3a* mutant alleles that all lack the homeodomain. Embryos homozygous for two of these alleles develop abnormally and die, whereas zebrafish homozygous for the other two alleles are viable. We found that all four mutations abrogate binding to Prmt2 and Tle3b, whereas Azin1b binding was preserved in all cases. Interestingly, Hmgb1a and Hmgn3 had more affinity for products of the viable mutant alleles. These data shed light on how HMX3/Hmx3a might function at a molecular level and identify new targets for future study in these vital developmental processes.

HMX3 is an NKX-family protein^1,2^ with several essential functions during vertebrate development. For example, in both zebrafish and mouse, Hmx3a and HMX3, respectively, are required for correct ear development^3–7^. Loss of *Hmx3/hmx3a* results in fusion of the utricle and saccule in the inner ear. The utricle detects linear acceleration and tilt in the horizontal plane and coordinates auditory function and the saccule detects linear acceleration and tilt in the vertical plane and coordinates vestibular function^3–5^. In zebrafish, the fusion of these two sensory inner ear organs also manifests as a fusion of the overlying utricular and saccular otoliths^3–5^. In zebrafish, *hmx3a* is also required for correct development of the lateral line mechanosensory system^4,5^ and for a subset of dI2 spinal cord interneurons to adopt their correct neurotransmitter phenotype^5^. As mouse *Hmx3* was recently shown to be expressed in equivalent spinal cord neurons^8^, the latter function is likely conserved between these species. In mouse, *Hmx3* is also required for uterine implantation of embryos and is redundantly required with the related gene, *Hmx2*, for specification of the hypothalamus^3,6^. Zebrafish also have an *hmx2* gene that has a similar expression pattern to *hmx3a* but, surprisingly, this gene does not seem to be required for any of these aspects of embryonic development^5^. Zebrafish also have a teleost-specific duplicate *hmx3* gene, *hmx3b*, but this gene is not expressed at the appropriate times or places to be involved in these fundamental developmental processes^5^. While mouse *Hmx3* mutants are viable to adulthood^3^, zebrafish embryos that completely lack Hmx3a are embryonically lethal^5^. Given these crucial roles of HMX3/Hmx3a in vertebrate development, it is vital that we identify the molecular interactions and mechanisms necessary for HMX3/Hmx3a function at a molecular level.

As mentioned above, Hmx3a belongs to the NKX family of proteins, all of which contain a DNA-binding domain called a homeodomain^1,2^^.^ In our previous investigation of *hmx3a* functions in embryonic development, we created several *hmx3a* indel mutant lines^5^, including *hmx3a*^*SU3*^, *hmx3a*^*SU43*^, *hmx3a*^*sa23054*^, and *hmx3a*^*SU42*^. Embryos homozygous for *hmx3a*^*SU3*^ or *hmx3a*^*SU43*^ alleles, which should both encode Hmx3a proteins that truncate prior to the homeodomain, have abnormal spinal cord, lateral line, and ear phenotypes and are embryonically lethal^5^. Spinal cord neurotransmitter phenotypes of *hmx3a*^*SU3*^ and *hmx3a*^*SU43*^ homozygotes are just as severe as those of deletion mutants that lack all *hmx3a* coding sequence (*hmx2;hmx3a*^*SU44*^ and *hmx2;hmx3a*^*SU45*^)^5^, indicating that they are likely null alleles. However, embryos homozygous for *hmx3a*^*sa23054*^ or *hmx3a*^*SU42*^ alleles, which should also encode truncated Hmx3a mutant proteins which lack the homeodomain and have very similar amounts of N-terminal wild-type (WT) sequence compared to *hmx3a*^*SU3*^ and *hmx3a*^*SU43*^, do not have these abnormal phenotypes (with the exception that some *hmx3a*^*sa23054*^ homozygotes have variable phenotypes) and they grow up into fertile adults^5^. Importantly, we did not detect genetic compensation in *hmx3a*^*SU42*^ mutants, suggesting that this is not the reason for the lack of abnormal phenotypes in these embryos. Taken together, these data suggest the intriguing hypothesis that Hmx3a may not require its homeodomain for its essential roles in embryonic development or viability^5^.

These results were unexpected and raised important questions: First, if Hmx3a does not need its DNA-binding domain and, therefore, does not act as a classically-defined transcription factor, what is the molecular mechanism(s) through which it functions in these crucial developmental processes? Second, why do these very similar mutant alleles have such different phenotypic consequences? Prior to this study, the homeodomain was the only functional domain that had been identified in Hmx3/Hmx3a and importantly, no binding partners for these proteins had ever been identified, so there were no clues as to how HMX3/Hmx3a might function in the absence of the homeodomain. Though we do not know for certain that all the mutant-encoded proteins are translated in vivo or whether they are degraded once they are translated*,* all of these mutant alleles still express mRNA, and this mRNA is not subject to nonsense-mediated decay^5^. While each of these mutant alleles introduces a frameshift or an immediate stop codon and should encode a truncated protein with a similar N-terminal length of WT amino acid residues, the number of non-WT residues prior to the new stop codon varies. Therefore, we hypothesized that the differences between mutant phenotypes might reflect whether the mutant proteins can still bind specific protein binding partners.

To identify proteins that interact with HMX3/Hmx3a, we performed a yeast two-hybrid (Y2H) screen with full-length mouse HMX3 and a mouse E11 cDNA library, because of the lack of appropriately staged, commercially available zebrafish Y2H libraries. Given the high sequence and functional conservation between mouse HMX3 and zebrafish Hmx3a, we hypothesized that important protein interactions would be conserved between these two species. We sequenced plasmids encoding putative binding partners from over 3000 positive colonies using high throughput methods and ultimately identified 539 unique proteins. We prioritized a subset of these putative binding partners for further analyses based on their expression, conservation, intracellular localization, and known functions. We cloned the zebrafish orthologs of these proteins as Glutathione S-Transferase (GST)-fusions. We then used Co-Immunoprecipitation (Co-IP) experiments to test whether these proteins bind to recombinant full-length zebrafish Hmx3a. We confirmed that the protein Tle3b binds Hmx3a and performed deletion analysis to map the interaction site, finding that the WDR-domain of Tle3b binds to the C-terminus of Hmx3a and not to the canonically-predicted Tle-binding motifs that we identified bioinformatically. We also demonstrated that zebrafish Hmx3a interacts with Prmt2, Azin1b, Hmgn3, and Hmgb1a. We tested whether proteins that bind full-length Hmx3a also bind the truncated Hmx3a proteins encoded by *hmx3a*^*SU3*^, *hmx3a*^*SU43*^, *hmx3a*^*sa23054*^, and *hmx3a*^*SU42*^ mutant alleles. We found that while Azin1b binds all four mutant Hmx3a proteins, Prmt2 and Tle3b do not bind any of them, and Hmgb1a and Hmgn3 have more nuanced interaction profiles, with more pronounced binding to the products of *hmx3a* alleles that do not produce obvious abnormal phenotypes in vivo. Taken together, these findings provide crucial information about putative binding partners and functional domains of HMX3/Hmx3a and identify proteins that may have important roles in embryonic development.

## Results

### Isolation of novel binding partners of HMX3 with yeast two-hybrid (Y2H) screening

We first wanted to identify the oligomeric status of functional HMX/Hmx proteins. To do this, we tested whether HMX3/Hmx3a and/or the closely related proteins HMX2/Hmx2 homo-dimerize or hetero-dimerize to each other by cloning mouse *HMX3* and *HMX2* and zebrafish *hmx3a* and *hmx2* into both the pGBKT7-BD bait vector and the pGADT7-AD prey vector from the Clontech Matchmaker® Gold Y2H system. We initially tested all of these constructs individually for expression, autoactivation, and toxicity. We crossed all bait constructs with all prey constructs, and no interactions were detected between any HMX3/Hmx3a and/or HMX2/Hmx2 protein pair (data not shown). These results suggest that these proteins function as monomers and not as homo- or heterodimers.

**Figure 1:**
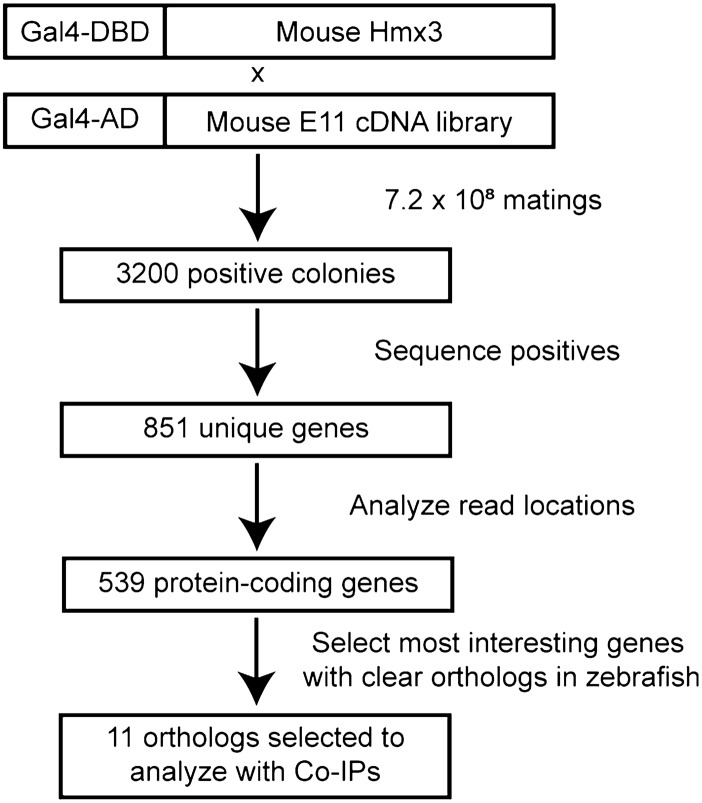
Isolation of novel binding partners of HMX3 with yeast two-hybrid screening. Top: Schematic representation of bait and prey constructs used in screening. Full-length mouse HMX3 fused to the Gal4 DNA-binding domain (DBD) was used as bait. A library of stage E11 mouse cDNAs fused to the Gal4 activating domain (AD) was used as prey. 7.2 × 10^8^ solid plate matings were performed, yielding 3200 positive colonies. Positive prey inserts were segregated and sequenced and reads were aligned to the mouse genome, identifying 851 unique genes. After removing non-coding genes and those for which only untranslated region (UTR) or intronic sequences were recovered, 539 protein-coding sequences remained. Zebrafish orthologs of the most functionally interesting genes were selected for further analysis with Co-IPs.

Having established that mouse HMX3 and zebrafish Hmx3a do not self-associate and also do not associate with HMX2/Hmx2, we next performed a large-scale screen to identify novel binding partners of HMX3 (Fig. 1). All the data so far suggest that most functions of HMX3/Hmx3a are conserved between different vertebrates^3–7^. Therefore, as there were no appropriately staged, commercially available zebrafish Y2H libraries and we were most interested in identifying protein interactions that are conserved between species, we used mouse HMX3 as a bait protein and a mouse E11 cDNA library (Clontech Mate & Plate Mouse E11 Day Library) for the prey proteins. Using solid plate mating, we screened 7.2 × 10^8^ diploid colonies on *AUR1-C* reporter plates. 3200 (0.000004%) of these crosses yielded positive colonies. We replica plated these colonies to verify their positive interaction status, before lysing them, amplifying the prey inserts with PCR, sequencing the inserts, and aligning the read sequences to the mouse genome. In this way, we identified 539 unique genes with reads aligned specifically to protein-coding sequence (see Supplementary Table S1 online). A subset of these, including prioritized candidates (labeled with * or **) and the most abundant sequences as determined by read count (see Supplementary Table S1 online) are listed in Table 1.

**Table 1:** Subset of HMX3 binding partners identified with yeast two-hybrid screen. Subset shown here includes prioritized genes (* or **) and other most abundant genes identified by sequencing read count (columns 1, 3 and 5). * indicates genes for which zebrafish orthologs were confirmed to bind full-length zebrafish Hmx3a using Co-IPs. ** indicates genes for which zebrafish orthologs did not bind full-length zebrafish Hmx3a in Co-IPs. Columns 2, 4 and 6 show unique MGI Accession IDs for each gene.

Gene Symbol	MGI Gene/Marker ID	Gene Symbol	MGI Gene/Marker ID	Gene Symbol	MGI Gene/Marker ID
Prmt2*	MGI:1,316,652	*Flna*	MGI:95,556	*Inpp5k*	MGI:1,194,899
Azin1*	MGI:1,859,169	*Tceal9*	MGI:109,567	*Prmt7*	MGI:2,384,879
Tle4*	MGI:104,633	*Emilin1*	MGI:1,926,189	*Gnb4*	MGI:104,581
Hmgb1*	MGI:96,113	*Hmgxb3*	MGI:2,441,817	*Cope*	MGI:1,891,702
Hmgn3*	MGI:2,138,069	*Ahcy*	MGI:87,968	*Ccpg1os*	MGI:3,648,770
Fez1**	MGI:2,670,976	*Ndufa9*	MGI:1,913,358	*Fip1l1*	MGI:1,914,149
Wdr61**	MGI:1,917,493	*Pias1*	MGI:1,913,125	*Cops5*	MGI:1,349,415
Rack1**	MGI:101,849	*Atp5a1*	MGI:88,115	*Rpa1*	MGI:1,915,525
Calr**	MGI:88,252	*Jam3*	MGI:1,933,825	*Nup43*	MGI:1,917,162
Sdcbp**	MGI:1,337,026	*Dram2*	MGI:1,914,421	*Efcab7*	MGI:2,385,199
Oaz1**	MGI:109,433	*Sars*	MGI:102,809	*Hnrnph1*	MGI:1,891,925
*Ube2i*	MGI:107,365	*Hmgn2*	MGI:96,136	*Psmc5*	MGI:105,047
*Eef2*	MGI:95,288	*Cnot6*	MGI:2,144,529	*Ctse*	MGI:107,361
*Myl6*	MGI:109,318	*Ptpn9*	MGI:1,928,376	*Rnf2*	MGI:1,101,759
*Itm2a*	MGI:107,706	*Orc3*	MGI:1,354,944	*Actc1*	MGI:87,905
*Sec11a*	MGI:1,929,464	*Ube2e1*	MGI:107,411	*Tulp4*	MGI:1,916,092
*Pcca*	MGI:97,499	*Dctn6*	MGI:1,343,154	*Ndufa1*	MGI:1,929,511
*Cnn3*	MGI:1,919,244	*Smchd1*	MGI:1,921,605	*Tmem176b*	MGI:1,916,348
*Eef1a1*	MGI:1,096,881	*Ivns1abp*	MGI:2,152,389	*Kcnk10*	MGI:1,919,508
*Pcbp1*	MGI:1,345,635	*Polr1b*	MGI:108,014	*Fbln1*	MGI:95,487
*Mrpl4*	MGI:2,137,210	*Abcb10*	MGI:1,860,508	*Safb2*	MGI:2,146,808
*Eif1a*	MGI:95,298	*Sccpdh*	MGI:1,924,486	*Rbbp7*	MGI:1,194,910
*Npc2*	MGI:1,915,213	*Metap2*	MGI:1,929,701	*Lpar1*	MGI:108,429
*Tuba1b*	MGI:107,804	*Nsmaf*	MGI:1,341,864	*Robo3*	MGI:1,343,102
*Psma6*	MGI:1,347,006	*Trim3*	MGI:1,860,040	*Ldha*	MGI:96,759
*Eno1b*	MGI:3,648,653	*Col18a1*	MGI:88,451	*Hdhd3*	MGI:1,919,998
*Leng1*	MGI:1,917,007	*Ccdc66*	MGI:2,443,639	*Tysnd1*	MGI:1,919,017
*Timm17b*	MGI:1,343,176	*Nit2*	MGI:1,261,838	*Camk1g*	MGI:2,388,073
*Fkbp8*	MGI:1,341,070	*Papss1*	MGI:1,330,587	*Srsf3*	MGI:98,285
*Rpl5*	MGI:102,854	*Pgm2*	MGI:97,564	*Orc5*	MGI:1,347,044
*Bpgm*	MGI:1,098,242	*Asf1b*	MGI:1,914,179	*Hdac2*	MGI:1,097,691
*Srsf2*	MGI:98,284	*H3f3b*	MGI:1,101,768	*Zmym4*	MGI:1,915,035
*Dnajc4*	MGI:1,927,346	*Khsrp*	MGI:1,336,214	*Prdx2*	MGI:109,486
*Plcg1*	MGI:97,615	*Eif3a*	MGI:95,301	*Cfap91*	MGI:2,443,598
*Actg1*	MGI:87,906	*Psmb1*	MGI:104,884	*Arhgap29*	MGI:2,443,818
*Banp*	MGI:1,889,023	*Zfand2b*	MGI:1,916,068	*Mrpl39*	MGI:1,351,620
*Vim*	MGI:98,932	*Taf1d*	MGI:1,922,566	*Tspan3*	MGI:1,928,098
*Commd4*	MGI:1,913,449	*Psmc3*	MGI:1,098,754	*Gfm2*	MGI:2,444,783
*Ahcyl*	MGI:3,643,647	*Lat2*	MGI:1,926,479	*Uba3*	MGI:1,341,217
*Tmem167*	MGI:1,913,324	*Sdhb*	MGI:1,914,930	*Ptpn2*	MGI:97,806

We excluded from further analyses genes that either had no obvious ortholog in zebrafish or that were likely to be Y2H false positives because they, for example, encoded proteasome components, cytoskeletal components, or mitochondrial proteins^9^. Based on expression, conservation, intracellular localization, and known functions, we selected 11 distinct putative binding partners from this filtered list (RACK1, SDCBP, WDR61, FEZ1, CALR, OAZ1, PRMT2, AZIN1, HMGN3, HMGB1, and TLE4) to further analyze using the closest zebrafish orthologs, which we cloned as expression constructs. Most of these mouse proteins had single, unambiguous orthologs in zebrafish. In cases where zebrafish have duplicate genes (*Hmgb1*: *hmgb1a* and *hmgb1b*; *Azin1*: *azin1a* and *azin1b*; *Oaz1*: *oaz1a* and *oaz1b*) or two otherwise close orthologs (*Tle4*: *tle3a* and *tle3b*), we cloned the ortholog with the highest amino acid conservation compared to the mouse protein.

### Prmt2, Azin1b, Hmgn3, Hmgb1a, and Tle3b bind full-length Hmx3a in Co-IPs

**Figure 2:**
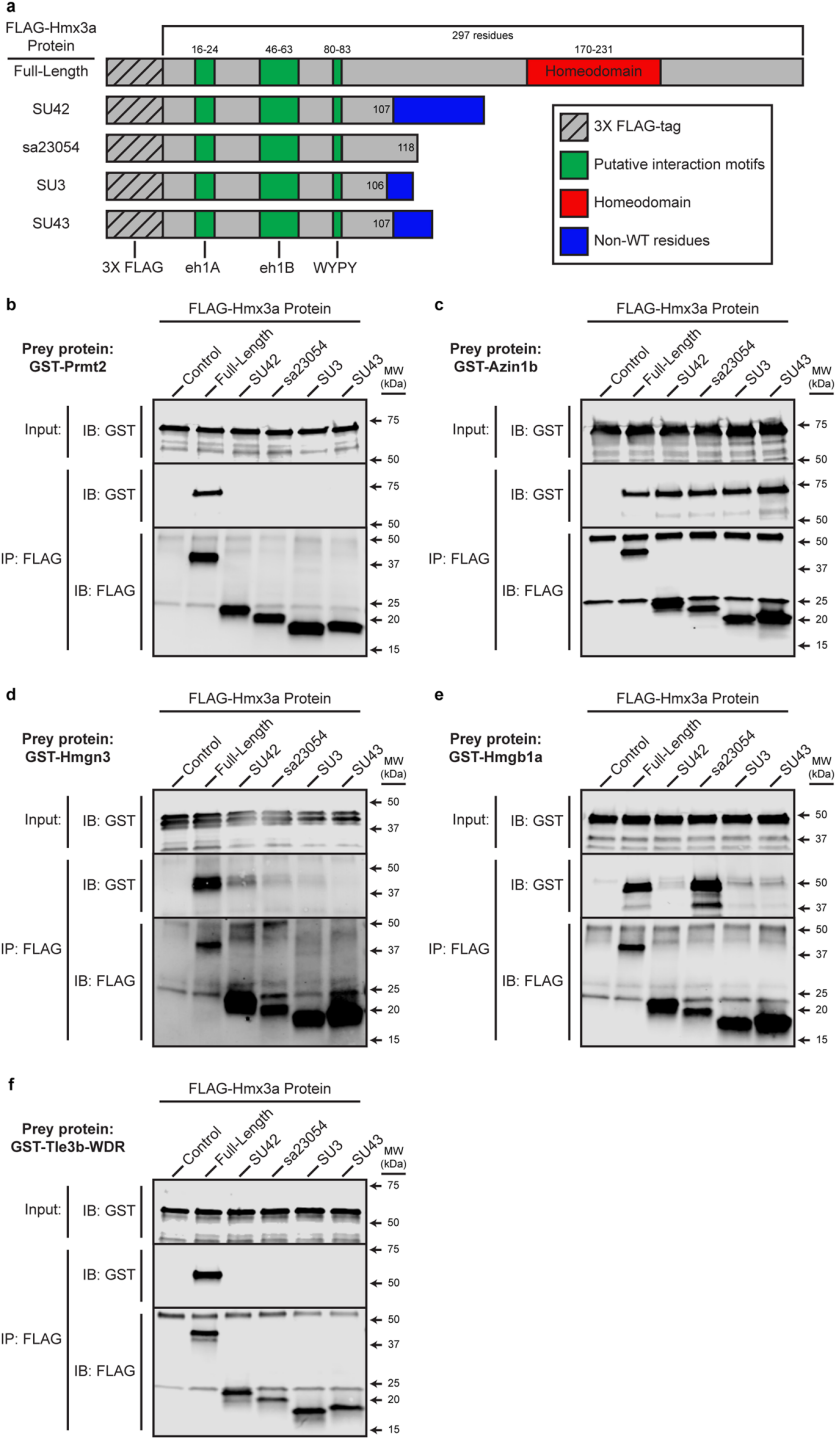
Zebrafish Prmt2, Azin1b, Hmgn3, Hmgb1a, and the WDR domain of Tle3b bind full-length zebrafish Hmx3a in Co-IPs and have varying interaction profiles with proteins encoded by *hmx3a* mutant alleles. (**a**) Schematics of FLAG-tagged Hmx3a constructs used in these experiments. SU42, sa23054, SU43, and SU3 indicate different mutant forms of Hmx3a (See ^5^). eh1A, eh1B, and WYPY indicate predicted interaction motifs for Tle3b-WDR. Numbers indicate residues encompassing annotated domains or the total number of WT residues in each protein. (**b**–**f**) Putative binding partners were expressed as GST-fusion proteins, purified, and incubated with either a FLAG-Hmx3a-expressing embryonic lysate or a stage-matched control lysate expressing no recombinant Hmx3a. Proteins were immunoprecipitated with an anti-FLAG antibody and separated by SDS-PAGE followed by transfer to a membrane and immunoblotting with anti-FLAG or anti-GST antibody. Top images in each panel show anti-GST immunoblot (IB) of 4% of input. Middle and bottom images in each panel show blots for GST and FLAG, respectively, of the immunoprecipitate (IP). The Hmx3a protein included in each lysate is indicated along the top *x* axis. Molecular weight (MW) is shown on the right-hand side. Prey proteins were (**b**) Prmt2, (**c**) Azin1b, (**d**) Hmgn3, (**e**) Hmgb1a, and (**f**) the WDR domain of Tle3b. FLAG antibody heavy and light chains appear at just above 50 and at 25 kDa, respectively in IP:FLAG;IB:FLAG fractions. Non-specific signal from the FLAG antibody heavy chain is also slightly visible in IP:FLAG;IB:GST fractions in (**d**) and (**e**). In all cases, the highest MW band in anti-GST input fractions is the full-length GST fusion protein. n = at least 2 for all experiments. Original blots are presented in Supplementary Fig. S3.

To test whether these zebrafish orthologs bind zebrafish Hmx3a, we cloned full-length cDNA for *rack1*, *sdcbp*, *wdr61*, *fez1*, *calr*, *oaz1a* (closest ortholog of mouse OAZ1), *prmt2*, *azin1b* (closest ortholog of mouse AZIN1), *hmgn3*, and *hmgb1a* (closest ortholog of mouse HMGB1). *oaz1a* requires ribosomal frameshifting in vivo to bypass a single “T” nucleotide in the endogenous transcript in order to be translated in-frame in vivo. Therefore, we cloned this gene without that “T”, so Oaz1a could be translated in-frame in *E. coli*. Mouse TLE4, and its closest zebrafish ortholog Tle3b, are both large proteins: 83.787 kDa and 83.789 respectively. The only domain of TLE4 that we isolated in our Y2H screen was the C-terminal WD-Repeat (WDR) domain. Therefore, we only cloned this domain from Tle3b, as we were concerned that if we tried to express the full-length protein we might get translation products truncated ahead of the C-terminal WDR domain, or the protein might not express well or be soluable. We cloned all of these constructs as GST-fusions, expressed them in *E. coli*, purified them, and tested them in Co-IPs with FLAG-tagged Hmx3a-expressing zebrafish lysates, harvested from embryos microinjected with synthetic mRNA at the one-cell stage. As a control, we used lysates that were not expressing FLAG-tagged Hmx3a. GST-Rack1, -Sdcbp, -Wdr61, -Fez1, -Calr, and -Oaz1a did not bind full-length FLAG-Hmx3a in these Co-IPs (see Supplementary Fig. S1 online). In contrast, GST-Prmt2, -Azin1b, -Hmgn3, -Hmgb1a, and -Tle3b-WDR all bound full-length FLAG-Hmx3a (Fig. 2), confirming that these binding partners are not Y2H false-positives and that these protein interactions are conserved between mouse and zebrafish.

### Interaction profiles of proteins encoded by hmx3a mutant alleles differ from that of full-length Hmx3a

We next asked whether Prmt2, Azin1b, Hmgn3, Hmgb1a, and Tle3b-WDR also bind the truncated proteins encoded by *hmx3a*^*SU3*^, *hmx3a*^*SU43*^*, hmx3a*^*sa23054*^ or *hmx3a*^*SU42*^ mutant alleles (Fig. 2). We performed Co-IPs using recombinant, FLAG-tagged versions of these proteins that exactly match the proteins that should be encoded by each mutant sequence, including the C-terminal, non-WT amino acids introduced by each respective frameshift (Fig. 2a). We were particularly interested in determining whether any of these proteins bind the products of the viable Hmx3a mutant alleles but do not bind the products of the embryonic lethal mutant alleles, since this is what we would predict if a particular protein interaction is required for the developmental functions of Hmx3a.

In our Co-IPs, Prmt2 and Tle3b-WDR bound only full-length Hmx3a (Fig. 2b,f, respectively), suggesting they bind somewhere in the C-terminal portion of the protein that is lost in all mutants. In contrast, Azin1b bound all four truncated proteins (Fig. 2c), indicating that it binds somewhere in the N-terminal portion that is retained by all of the mutant proteins that we tested. Results were more nuanced for both Hmgn3 and Hmgb1a. At least a very small amount of each respective protein was co-precipitated by all four truncated Hmx3a baits. Hmgn3 bound all of the truncated Hmx3a proteins more weakly than full-length Hmx3a, but bound Hmx3a^SU42^ and, to a lesser extent, Hmx3a^sa23054^, more strongly than the other truncated proteins (Fig. 2d). Hmgb1a, however, bound Hmx3a^sa23054^ with similar affinity to full-length Hmx3a, whilst binding much more weakly to the other three truncated proteins (Fig. 2e).

We could not find any information that suggested where in Hmx3a Prmt2, Azin1b, Hmgn3 and Hmgb1a might bind, and deletion studies of all of these proteins were outside the scope of this study. In contrast, previous studies of Tle proteins suggested that Tle3b might bind Hmx3a through either an eh1 or a WRP(W/Y) domain. As discussed in the introduction, Hmx proteins belong to the Nkx family. Other Nkx proteins bind Tle protein WDR domains through a small, highly variable motif called an eh1 domain^10–12^. In contrast, Hes/Her family transcription factors bind Tle protein WDR domains via a different WRP(W/Y) small motif^13^. Neither of these motifs were previously annotated in Hmx3a except for one mention of a possible eh1 domain from residues 46–63 in a study by Bayramov and colleagues^14^. We identified another putative eh1 domain in residues 16–24 by BLASTing the consensus eh1 sequence from Smith and Jaynes^10^. We hereafter refer to these putative eh1 domains as eh1A (residues 16–24) and eh1B (residues 46–63; see Supplementary Fig. S2). Hmx3a also has a similar motif to WRP(W/Y) in residues 80–83, which consists of the amino acid sequence WYPY (Supplementary Fig. S2). However, all three of these motifs are fully retained in the four truncated Hmx3a mutant proteins that did not bind Tle3b-WDR, suggesting that these three motifs are not sufficient for Hmx3a to interact with the Tle3b-WDR domain.

### Tle3b binds the carboxy-terminus of Hmx3a

**Figure 3:**
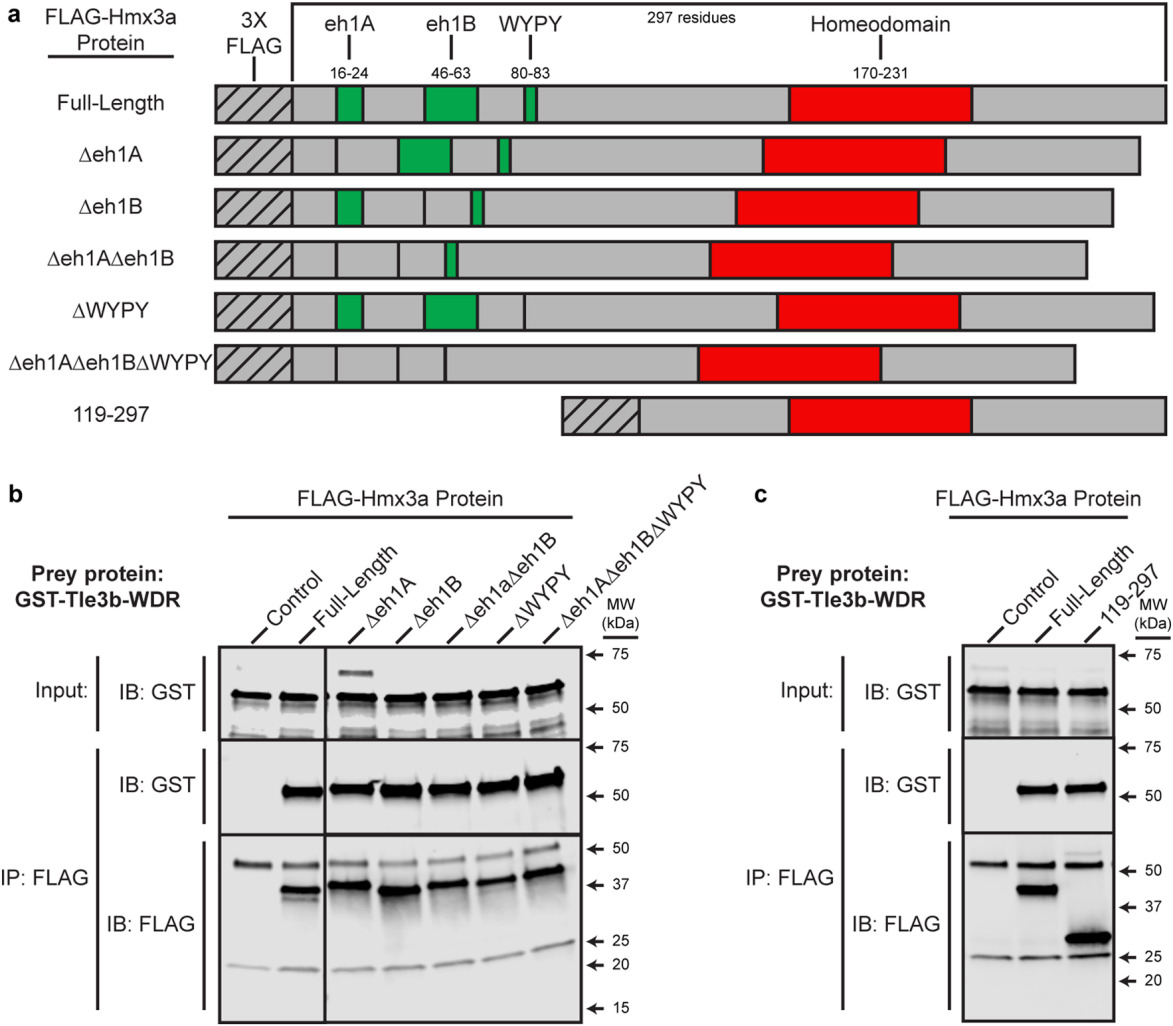
The WDR domain of Tle3b binds to the C-terminus of Hmx3a but not to any canonical Tle interaction motifs. (**a**) Schematics of FLAG-tagged Hmx3a constructs used in these experiments. eh1A, eh1B, and WYPY indicate predicted interaction motifs for Tle3b-WDR. Numbers (top) indicate residues encompassing annotated domains or (left-hand side, bottom row) included in the C-terminal construct. (**b**, **c**) The WDR domain of Tle3b was expressed as a GST-fusion protein, purified, and incubated with either a FLAG-Hmx3a-expressing embryo lysate or a stage-matched control lysate expressing no recombinant Hmx3a. Proteins were immunoprecipitated with an anti-FLAG antibody and separated by SDS-PAGE followed by transfer to a membrane and immunoblotting with anti-FLAG or anti-GST antibody. Top images in each panel show anti-GST immunoblot (IB) of 4% of input. Middle and bottom images in each panel show blots for GST and FLAG, respectively, of the immunoprecipitate (IB). MW is shown on the right-hand side. (**b**) Co-IPs with putative interaction motif deletion constructs. Blot images on each side of respective rows were from the same membrane and were processed identically for brightness/contrast. (**c**) Co-IP with carboxy-terminal Hmx3a construct. FLAG antibody heavy and light chains appear at just above 50 kDa and at 25 kDa, respectively in IP:FLAG;IB:FLAG fractions. Note that though it lacks 9 amino acids vs full-length Hmx3a, FLAG-Hmx3a-Δeh1A runs slightly above full-length Hmx3a (panel (**b**) IP:FLAG;IB:FLAG). A non-specific band, likely corresponding to the yolk protein Vitellogenin, is also visible above GST-Tle3b-WDR in the anti-GST input fraction of Δeh1A and, very slightly, in the control in panel (**b**). n = at least 2 for all experiments. Original blots are presented in Supplementary Fig. S3.

Although Tle3b-WDR did not bind any of the truncated Hmx3a proteins that we tested, we hypothesized that one or more of these canonical binding motifs might be required, in combination with a carboxy-terminal element, for Tle3b-WDR to bind full-length Hmx3a. Therefore, we created deletion FLAG-Hmx3a constructs that contain all of the Hmx3a sequence except one or other of the putative eh1 domains, the WYPY motif, both putative eh1 domains, or all three motifs (Fig. 3). However, we found that Tle3b-WDR bound all of these constructs with similar affinity to full-length Hmx3a (Fig. 3b), demonstrating that these motifs are not required for this interaction.

Since we had established that Tle3b-WDR does not bind to any of these canonical, N-terminal motifs, we tested whether the carboxy-terminus of Hmx3a is sufficient to interact with Tle3b-WDR. We cloned a FLAG-tagged version of residues 119–297 of Hmx3a, which comprises everything downstream of the last WT amino acid retained by any of the four mutant alleles. We found that Tle3b-WDR bound this C-terminal Hmx3a construct with similar affinity to full-length Hmx3a (Fig. 3c). Taken together, these data suggest that the binding site for Tle3b-WDR lies within residues 119–297 of Hmx3a and that the N-terminus of Hmx3a, which includes all previously hypothesized Tle-WDR binding sites, is completely dispensable for this interaction.

## Discussion

In this study, we identified 539 putative protein-binding partners of mouse HMX3 in a Y2H screen, including the subset prioritized for further analysis, RACK1, SDCBP, WDR61, FEZ1, CALR, OAZ1, PRMT2, AZIN1, HMGN3, HMGB1, and TLE4. We established that zebrafish orthologs Prmt2, Azin1b, Hmgn3, Hmgb1a, and Tle3b bind to zebrafish Hmx3a in Co-IPs, whereas Rack1, Sdcbp, Wdr61, Fez1, Calr, and Oaz1a do not. There are a variety of reasons why the latter six proteins might not bind Hmx3a in this assay. First, they may have been false positives in the Y2H screen. Second, these proteins might interact in mouse but not in zebrafish. Finally, the bacterial expression system used to generate these proteins, or the Co-IP assay itself, might preclude detection of physiologically relevant interactions. For example, the proteins may lack necessary post-translational modifications that bacteria do not add or there may be steric hindrance from the GST- or FLAG-tags. In addition, the end-point readout of western blot after several washes in buffer is not suitable for detection of weak or transient interactions. Therefore, we cannot unequivocally conclude that these proteins do not interact with Hmx3a in zebrafish. However, our data strongly suggest that Prmt2/PRMT2, Azin1b/AZIN1, Hmgn3/HMGN3, Hmgb1a/HMGB1, and Tle3b/TLE4 physically interact with Hmx3a/HMX3. Since we performed our Co-IPs using zebrafish embryo lysates, these experiments do not distinguish between direct or indirect interactions. However, as we originally identified these binding partners in a Y2H system, in which the only other proteins present are nuclear yeast proteins, they are likely to be direct. Importantly, the genes that encode all of these proteins are also expressed at appropriate developmental stages in tissues where HMX3/Hmx3a have essential functions, as would be expected for physiologically relevant binding partners^15–21^.

Given that we know so little about how Hmx3a/HMX3 performs its essential developmental functions, the binding partners that we have identified may reveal important clues about the molecular mechanisms of Hmx3a/HMX3 function. In addition, it is possible that the variation in viability, and in ear, CNS, and lateral line phenotypes observed in distinct zebrafish *hmx3a* mutants may be caused by some, but not all, mutant alleles no longer being able to interact with specific binding proteins. If this is the case, then proteins that bind the products of alleles with only subtle or no abnormal phenotypes, but not the products of alleles with severe phenotypes, would be good candidates for being required for the functions of Hmx3a in embryonic development and viability. However, as discussed in the introduction, it is also possible that the different phenotypes caused by these mutant alleles instead reflect the amount of truncated Hmx3a protein that is made and/or retained in each case. All four mutant alleles still express stable mRNA^5^, but we do not know whether these mRNAs are all translated or whether any of the mutant protein products are degraded once they are made. We attempted to assay this in several different ways in our previous study^5^, but a lack of specific antibodies, coupled with low expression of Hmx3a meant that we were unable to do so. However, if it is the case that the alleles that cause more severe phenotypes do so because there is less mutant protein present, any protein that still binds to the mutant Hmx3a proteins may have an important role in Hmx3a functions during development. In contrast, proteins that do not bind any of the truncated alleles are unlikely to be required for these functions, although they may still be important for as-yet-unidentified Hmx3a functions, for example in adult animals.

Since Azin1b bound all four truncated Hmx3a proteins, the phenotypical differences between *hmx3a* mutants cannot be due to this interaction being disturbed in some mutants and not others. However, given this retained binding and the ubiquitous expression of *azin1b* during embryogenesis^18,19,21^, it is still possible that Azin1b is required for Hmx3a functions during embryonic development. The principal function of Azin1b is to positively regulate the activity of the enzyme Odc1^22–24^, which is the rate-limiting enzyme in the anabolism of small molecules called polyamines. Polyamines play myriad roles in the cell and are involved in proliferation, survival, and differentiation^25^. They also bind to and modulate the activity of neurotransmitter receptors and other ion channels^26–28^. Since neuronal electrical activity can influence maintenance of neurotransmitter phenotypes^29,30^, it is possible that polyamines might also influence neurotransmitter phenotype maintenance through this mechanism.

Azin1b indirectly regulates Odc1, by binding to Oaz proteins. Oaz proteins can bind to Odc1 and target it for degradation^31,32^. Azin proteins structurally resemble Odc1 and have roughly a tenfold higher affinity for Oaz proteins than Odc1^22,23,33^. Unlike Odc1, Azins are not targeted for degradation by Oaz proteins so when they bind to Oaz proteins, they prevent them from binding to Odc1 and hence inhibit degradation of Odc1 and in effect increase synthesis of polyamines^33^. Interestingly, we also identified the Oaz protein OAZ1 as a putative binding partner of HMX3 in our Y2H screen, although we did not detect an interaction between zebrafish orthologs Oaz1a and Hmx3a via Co-IPs.

Since polyamines have so many different roles in the cell, it would be difficult to experimentally manipulate their levels and distinguish a specific effect on neurotransmitter phenotypes, distinct from a defect in neuron proliferation, survival, or differentiation. However, it would be interesting to test, in future studies, whether levels of polyamines are altered when Hmx3a is either experimentally depleted or overexpressed.

In addition to its role in polyamine regulation, mouse AZIN1 also binds the transcription factor DDX1 and cooperates with it to drive hematopoietic stem cell differentiation^34^. ChIP experiments identified AZIN1 residence at DDX1-target genomic loci and overexpression of AZIN1 led to increased expression of DDX1-target genes^34^. This suggests that AZIN1 can act in transcriptional complexes, independent of its function regulating polyamine metabolism. Therefore, it would also be interesting to assess, in future studies, whether HMX3/Hmx3a cooperate with AZIN1/Azin1b in transcriptional regulation.

In contrast to Azin1b, our Co-IP data suggest that Prmt2 does not bind any of the Hmx3a mutant proteins that we analyzed. This suggests that its interaction with Hmx3a is not required for any of the aspects of development that still occur normally in *hmx3a*^*SU42*^ or *hmx3a*^*sa23054*^ homozygous mutants. However, we cannot rule out the possibility that Prmt2 is required for other functions of Hmx3a that we did not analyze, for example in adult animals. Prmt2 is a protein methyltransferase, and it associates with transcription factors and methylates histone proteins at target loci^35,36^. For example, Prmt2 is essential in *Xenopus* for establishing the dorsal developmental program in response to Wnt signaling (an important pathway in many aspects of development^37^) through its association with β-catenin^35^. Therefore, it is possible that Prmt2 might methylate Hmx3a and/or that Hmx3a might transport Prmt2 to target promoters.

Tle3b-WDR also did not bind any of the mutant Hmx3a proteins. As with Prmt2, this suggests that Hmx3a does not need to bind Tle3b in order to function in any of the aspects of development that still occur normally in *hmx3a*^*SU42*^ or *hmx3a*^*sa23054*^ homozygous mutants. However, it is still possible that Tle3b may be required for other functions of Hmx3a that we did not analyze. Tle3b belongs to the Tle/Groucho family of transcriptional co-repressors, which form homo- or hetero-tetramers and are thought to act redundantly with one another^38,39^. Interestingly, many transcription factors that are important for patterning the spinal cord bind Tle proteins, including Tlx1 and Tlx3^40^ which, prior to our recent analyses of Hmx3a, were the only transcription factors implicated in specifying glutamatergic fates in dorsal spinal cord neurons^41,42^. Tle proteins also have an important role in modulating Wnt signaling. In the absence of active Wnt signaling, Tle proteins bind Tcf/Lef transcription factors and repress Wnt target genes^38^. Another homeodomain-protein, Lbx2, activates Wnt signaling by binding and sequestering Tle proteins^43^. This raises the intriguing possibility that Hmx3a might similarly sequester Tle proteins. It is also interesting that in humans, multiple Tle proteins bind Hmgb1^44^, as this suggests that Hmgb1a and Tle3b could form a complex with Hmx3a.

To our surprise, we found that the putative canonical Tle-binding motifs we identified in Hmx3a were dispensable for its interaction with Tle3b-WDR, and that, instead, the carboxy-terminal portion of Hmx3a after WT residue 119 was both required and sufficient for this interaction. This is important as it reveals a novel way that Tle proteins can bind other proteins. There are no putative eh1 or WRP(W/Y) motifs in this region of Hmx3a. WRP(W/Y) motifs have, to date, only been described at the C-terminus of Hes/Her proteins^45,46^. Intriguingly, the last four residues of Hmx3a are LRPV, which has slight similarity to WRP(W/Y). Therefore, it would be interesting to test, in future experiments, if these amino acids are required for the interaction of Tle3b-WDR with Hmx3a. It would also be interesting to perform deletion, and if possible motif, analyses of the other proteins that bind Hmx3a, to narrow down the interaction domains.

In contrast to the other proteins discussed above, our Co-IP experiments suggest that Hmgn3 binds all of the mutant Hmx3a proteins that we tested more weakly than full-length Hmx3a. However, it binds the two alleles that cause either variable, partially penetrant or no abnormal embryonic phenotypes more strongly than the alleles that result in fully penetrant, severe abnormal phenotypes. Our results also suggest that Hmgb1a binds Hmx3a^sa23054^ with similar affinity to full-length Hmx3a, but that it binds the other three truncated proteins more weakly. These data suggest that the interactions of these proteins with Hmx3a could partially explain the differential *hmx3a* mutant phenotypes. Hmgn3 and Hmgb1a are members of two separate superfamilies of High Mobility Group (HMG) proteins. HMG proteins are small DNA-binding proteins involved in transcription, replication, recombination, and DNA-repair^47^. Hmgn3 has a nucleosome-binding domain and belongs to the Hmgn family, whereas Hmgb1a has two HMG-box DNA-binding domains and belongs to the Hmgb family. Proteins from both families bind transcription factors and facilitate their interaction with DNA and regulation of target gene expression^48,49^. Since Hmx3a may not need its own DNA-binding domain to function^5^, it is intriguing that it binds to these DNA-binding factors. It is also interesting that the products of *hmx3a*^*SU42*^ and *hmx3a*^*sa23054*^ mutant alleles retained affinity for GST-Hmgn3 and GST-Hmgb1a, respectively, since this could represent a mechanism through which these proteins remain functional in vivo.

Little is known about zebrafish *hmgn3*, other than it is highly expressed in the otic vesicle and CNS during embryogenesis^18^. In mice and humans, *HMGN3* is highly expressed in at least the developing CNS and eye^15,19,50^ and adult pancreatic islet cells^15^. Given the expression of *hmgn3* in the developing ear and CNS, it would be interesting to investigate whether, like *hmx3a*, it is required for otic vesicle development and correct neurotransmitter phenotype specification in the spinal cord.

Zebrafish have two orthologs of *Hmgb1, hmgb1a* and *hmgb1b*. Both are broadly expressed during embryogenesis but are specifically enriched in CNS and lateral line primordium^18,20^. While both remain abundant in the CNS through at least 48 hpf, only *hmgb1a* is still expressed in lateral line neuromasts at this stage^20^. Interestingly, in mouse, the related protein HMGB2 binds the Wnt effector transcription factor LEF1 and potentiates transcriptional activation of the LEF1-β-CATENIN complex^51^. Similarly, knocking down zebrafish Hmgb1a with translation-blocking morpholinos also alters Wnt signaling^52,53^.

It is intriguing that Hmx3a binds several Wnt-modulating proteins, especially given that Wnt signaling is essential for patterning the dorsal spinal cord^54^, proper otic vesicle development^55,56^, and morphogenesis of the lateral line^57^. It will be interesting to assess, in future experiments, whether Hmx3a cooperates with any of these proteins in regulating downstream effects of Wnt signaling, and whether this might be part of the mechanism through which Hmx3a carries out its essential developmental functions. As a first step, this could be tested by examining the effects of Hmx3a depletion or overexpression on established Wnt reporter lines like TOP-dGFP^58^.

In conclusion, we have identified 539 putative protein binding partners of mouse HMX3 and confirmed that at least five of these interactions are conserved in zebrafish. Our data suggest that confirmed binding partners Tle3b and Prmt2 may not be required for Hmx3a functions in ear, lateral line and spinal cord development or viability, but that Azin1b, Hmgb1a, and/or Hmgn3 may be important cofactors in these processes. Moreover, we found that Hmgb1a and Hmgn3 retain higher affinity for mutant Hmx3a proteins that retain WT functions than mutant proteins that result in abnormal development. This suggests that interactions with Hmgb1a and Hmgn3 may be required for Hmx3a functions during embryonic development. More broadly, the binding partners that we have identified offer important clues as to how Hmx3a might function and provide promising new targets for the study of CNS, ear, and lateral line development.

## Materials and methods

### Ethics statement

All zebrafish experiments in this research were approved by the Syracuse University Institutional Animal Care and Use Committee and performed in accordance with ARRIVE guidelines. All methods were carried out in accordance with relevant guidelines and regulations.

### Zebrafish husbandry and fish lines

Zebrafish (*Danio rerio*) were maintained on a 14 h light/10 h dark cycle at 28.5 °C. Embryos were obtained from paired and/or grouped spawnings of wild-type (WT; AB, TL, or AB/TL hybrid) adults.

### Yeast two-hybrid screen

We used the Matchmaker Gold Yeast Two-Hybrid system (630,489; Takara Bio). We cloned full-length mouse *Hmx3* as a fusion with *Gal4* DNA-binding domain in plasmid *pGBKT7* as bait, and transformed into Y2HGold Yeast Strain. We used mouse E11 cDNA library (630478; Takara Bio) cloned into *pGAD57* vector as prey, transformed into Y187 yeast strain. To detect bait:prey interactions, we mated bait and prey yeast strains, initially on 2 × YPDA plates for 24 h at 30 °C, before re-plating 720,000,000 crosses on YPD plates containing 125 ng/ml Aureobasidin A and incubating for 3 days at 30 °C. To confirm authenticity and stringency of bait:prey interactions, we patched all 3,200 positive colonies on to SD/-Leu/-Trp plates (to ensure growth of only colonies positive for both bait and prey) before replica plating on SD/-Leu/-Trp/-Ade, SD/-Leu/-Trp/-His and SD/-Leu/-Trp/X-α-Gal/AbA agar plates, which test all four assay reporters (X-α-Gal and Aureobasidin A (AbA) final concentrations: 40 µg/ml and 125 ng/ml respectively). We made glycerol stocks of each positive colony by scraping cells from a freshly patched SD/-Leu/-Trp plate into an individual well of a 96-well plate and adding 200 µl of 25% glycerol in YPD medium. Plates were sealed and vortexed before storing at −80 °C. We seeded new solid plate cultures on SPD/-Leu/-Trp from these glycerol stocks, grew them at 30 °C for 3 days, picked colonies and lysed them in 1.2 M sorbitol, 100 mM sodium phosphate, 200 U/ml β-glucuronidase pH 7.4 5 min at 37 °C. We used these lysates as templates for PCR amplification of prey inserts using primers and PCR conditions set 1 from Supplementary Table S2 (online). We purified prey insert amplicons using AMPure XP beads (A64881; Takara Bio) according to manufacturer’s instructions and analyzed concentration and size of each amplicon by gel electrophoresis with DNA mass standards (N0550; NEB). Amplicons were pooled at approximately equal molarity and a high-throughput sequencing library was prepared with a Nextera XT DNA Library Preparation Kit (FC-131-1096; Illumina). We sequenced this library with an Illumina MiSeq instrument (SY-410-1003; Illumina) using a MiSeq Reagent Nano Kit v2 (500 cycles) (MS-103-1003; Illumina).

We used Illumina’s native BaseSpace app “RNA-Seq Alignment” to generate a list of genes to which reads aligned. Sequences were aligned to the *Mus musculus* mm10 (RefSeq) mouse genome, using the TopHat (Bowtie2)^59^ algorithm and default parameter settings. Illumina’s “RNAReadCounter” utility was used to quantify numbers of reads aligned to each gene. These are gross numbers of reads and there was no length/abundance normalization or statistical analysis performed. The number of reads was used as a proxy for abundance of each amplicon within the master pool and the relative number of colonies containing sequence encoding each putative prey binding partner. The region(s) of each gene with sequencing coverage were manually investigated by importing alignment data into Integrative Genomics Viewer software^60^. Genes were annotated for whether protein-coding exons were covered and, if so, which exons were included. Candidates were prioritized using expression data obtained from ZFIN^18,61^ and Genepaint^19^, amino acid conservation based on sequences obtained from Ensembl^62^, intracellular localization information from Uniprot^63^, as well as functional information obtained from primary literature.

### Plasmid construction

All zebrafish Hmx3a expression plasmids were derived from a pCS2-based plasmid encoding 3XFLAG-tagged Hmx3a^5^. We used Q5 Site-Directed Mutagenesis PCR Kit (E0554S; NEB) to generate plasmids of WT Hmx3a with specific domains deleted or truncated versions encoded by *hmx3a* mutant alleles *SU42, sa23054, SU3* and *SU43*, including non-WT residues introduced into the coding sequence by these mutations (Figs. 2a, 3a). For primers and PCR conditions, see Supplementary Table S2.

We used a combination of Gibson Assembly (GST-Oaz1a) and Gateway® cloning (all other GST-fusions) to generate GST-fusion constructs of putative binding partners of Hmx3a. We isolated total RNA from 27 hpf WT embryos using TRIzol Reagent (15596018; Thermo Fisher Scientific) and RNeasy Mini Kit (74104; QIAGEN). Total RNA was converted to complementary DNA (cDNA) using iScript cDNA synthesis kit (1708891; Bio-Rad, Hercules, CA). We amplified sequence encoding each gene using Phusion polymerase (NEB M0530L; NEB) and primers in Supplementary Table S2. *Att*B sites for Gateway cloning and overlaps for Gibson Assembly were added to each amplicon via primer overhangs. We purified amplicons with EZ-10 Spin Column PCR Products Purification kit (BS664; Bio Basic). We recombined Gateway amplicons first into pDONR221 entry vector and subsequently into pDEST15 destination vector in a single tube using BP Clonase II (11789020; ThermoFisher Scientific) and LR Clonase II (11791020; ThermoFisher Scientific). *oaz1a* requires ribosomal frameshifting in vivo to bypass a single “T” nucleotide in the endogenous transcript in order to be translated in-frame in vivo. Therefore, in order to clone this gene without that “T”, we amplified the coding sequence 5’ and 3’ to this “T” in separate PCR reactions. These *oaz1a* amplicons were assembled into pDEST15 with an NEBuilder® HiFi DNA Assembly Cloning Kit (E5520S; NEB). Positive colonies were identified with colony PCR using primers and conditions from Supplementary Table S2. Minipreps were performed with a Plasmid Midi Kit (12143; QIAGEN) according to manufacturer’s instructions. Relevant portions of all plasmids were sequenced with Sanger sequencing (Genewiz) and primers in bold in Supplementary Table S2.

### Expression and recovery of recombinant, FLAG-tagged Hmx3a constructs

Plasmids encoding FLAG-tagged Hmx3a derivatives were linearized with NotI and mRNA was transcribed from 1 µg linearized plasmid with mMessage mMachine SP6 kit (AM1340; ThermoFisher Scientific). 3 nl of solution containing 1–2 ng of synthetic mRNA was injected into yolk of 1–4 cell stage WT embryos before incubating at 28.5 °C. After removing dead embryos, we harvested injected (or uninjected control) embryos for protein at 6 hpf. Embryos were enzymatically dechorionated by incubation in embryo medium + 1 mg/ml pronase (10,165,921,001; Sigma-Aldrich) for 10 min at room temperature, then transferred to a 1.5 ml Protein LoBind tube (022,431,081; Eppendorf) using a glass Pasteur pipette pre-coated with FBS. Embryos were quickly washed 6X with embryo medium to remove residual pronase, before adding 800 µl of ice-cold Ca^2+^-free Ringers Solution (116 mM NaCl, 2.9 mM KCl, 5.0 mM HEPES, pH 7.2) to each tube. This solution was removed and replaced with 800 µl of fresh Ca^2+^-free Ringers Solution. Embryos were mechanically de-yolked by vigorously pipetting up and down 4–6 times with a p1000 tip. Tubes were centrifuged at 300 g for 45 s to pellet cells. Supernatant was carefully removed, and cells were washed 4 times with ice-cold Ca^2+^-free Ringers Solution. Supernatant was again removed, and tubes were snap-frozen on dry ice before storing at −80 °C.

To lyse embryos, 1 µl of lysis buffer (50 mM Tris HCl, pH 8.0, 150 mM NaCl, 1% v/v Triton X-100, 100 µM PMSF (10837091001; Sigma-Aldrich), 0.125 µg/ml pepstatin A (Sigma-Aldrich, cat no. -P4265-5MG), 5 mM EDTA) per embryo was added to each tube before cells completely thawed. Cells were mechanically macerated with a micropestle. Tubes were incubated on ice for 10 min and centrifuged at 20,000 g for 1 min to pellet debris. The supernatant was carefully transferred to a new tube and the pellet was discarded. Clarified lysates were either analyzed immediately or snap-frozen on dry ice and stored at −80 °C. All lysates were analyzed by immunoblotting for FLAG prior to using them in Co-IPs to ensure concentrations of proteins of interest were similar across lysates.

### Expression and purification of GST-fusion proteins

Transformed *E. coli* of the strains NiCo21(DE3) (C2529; NEB), SHuffle T7 (C3026J; NEB), or Bl21-AI (C607003; ThermoFisher Scientific) were grown in Luria Broth (LB) medium at 37 °C until their optical densities (OD) reached 0.5–1.0. Cultures of NiCo21(DE3) cells or SHuffle cells were induced with 0.1–1.0 mM IPTG and cultures of Bl21-AI cells were induced with 0.2% w/v L-arabinose. After induction, cultures were either incubated at 37 °C for 1–4 h or moved to room temperature and grown overnight. Cells were harvested by centrifugation at 7000 g at 4 °C for 10 min. Supernatants were discarded and cell pellets were snap-frozen on dry ice before storing at −80 °C.

Cell pellets were resuspended in lysis buffer containing 50 mM Tris HCl, pH 8.0, 150 mM NaCl, 5 mM EDTA, 0.5 mg/ml human recombinant lysozyme (Sigma-Aldrich cat no. L1667-1G), 0.125 µg/ml pepstatin A, 500 µM PMSF, and 0.5% v/v Triton X100 (Sigma-Aldrich, cat no. X100-1L). Cells were kept on ice for 30 min, vortexed briefly while avoiding bubble formation, then incubated on ice for 20 min. Lysates were centrifuged at 7000 g at 4 °C for 30 min to pellet debris. Clarified lysates were decanted into new containers and snap-frozen on dry ice before storing at -80 °C.

GST-fusion proteins were purified in batches using glutathione (GSH) agarose beads (ThermoFisher, cat no. 16102BID). 400 µl GSH bead slurry was equilibrated with 10 ml wash buffer (50 mM Tris HCl, pH 8.0, 150 mM NaCl, 1 mM EDTA, 0.5% v/v Triton X100) in a 50 ml tube. Up to 50 ml lysate was added to each tube and incubated at room temperature for 30 min with end-over-end mixing. Beads were pelleted at 700 g for 3 min and then washed 2X 5 min with 50 ml wash buffer. GST-fusions were eluted four times with 1.5 ml volumes of elution buffer each time (wash buffer + 10 mM GSH (Sigma-Aldrich cat no. G4251-10G)) and concentration and purity were analyzed with SDS-PAGE.

### Co-immunoprecipitation (Co-IP), SDS-PAGE and Western blot analysis

Interactions between FLAG-tagged derivatives of Hmx3a and GST-fusions of putative binding partners were assayed with Co-IPs using Protein LoBind tubes (022431081; Eppendorf). All bait/prey combinations were tested in at least two independent experiments. Lysates of embryos expressing FLAG-Hmx3a constructs or uninjected, stage-matched negative control lysates were incubated with 5 µg of purified GST-fusion protein for 30 min at room temperature in 500 µl of wash buffer (50 mM Tris, pH 8.0, 150 mM NaCl, 1 mM EDTA, and 1% v/v Triton X-100). 45–60 µl of embryo lysate was used per tube, adjusted so that the amounts of each recombinant protein of interest were approximately equal. 60 µl of uninjected embryo lysate was used per control tube. 5 µl Protein G Dynabeads (10003D; ThermoFisher Scientific) were separately loaded with 2 µg FLAG antibody (F1804-200UG; Sigma-Aldrich) in 50 µl wash buffer + 100 ng/µl BSA and washed 2X with wash buffer to remove any unbound antibody. A magnetic rack was used to concentrate beads during all wash steps. Antibody-loaded beads were resuspended in 50 µl wash buffer per tube and dispensed into Co-IP tubes containing the FLAG-Hmx3a/control and GST-fusion mixture after setting aside 24 µl of this mixture as an input fraction. This final mixture was mixed end-over-end overnight at 4 °C then washed 4X with wash buffer. The resuspended beads were carefully transferred to new tubes after the second and fourth washes. After the final wash, beads were resuspended directly in 40 µl of freshly prepared 1X SDS-PAGE sample buffer (100 mM Tris HCl, pH 6.85, 4% w/v SDS, 0.2% w/v bromophenol blue, 20% v/v glycerol, 100 mM DTT).

Resuspended beads were heated to 95 °C for 5 min, then loaded directly into 4–20% Mini-PROTEAN® TGX™ Precast Protein Gels (4,561,095; Bio-rad). 20 µl of bead suspension was loaded for anti-GST blots and 10 µl was loaded for anti-FLAG blots. Input samples were mixed 1:1 with 2X SDS-PAGE sample buffer (200 mM Tris HCl, pH 6.85, 8% w/v SDS, 0.4% w/v bromophenol blue, 40% v/v glycerol, 200 mM DTT) and 20 µl of this suspension was loaded per lane. GST-fusion proteins were detected with either 1:1000 polyclonal rabbit anti-GST (CAB4169; Invitrogen) and 1:15,000 IRDye 800CW Goat anti-Rabbit IgG (925-32211; Licor) (Supplementary Fig. S1 panels a-f) or 1:1,000 monoclonal mouse GST antibody [Biotin], (A00867; Genscript) and 1:6,000 IRDye 800CW Streptavidin (925-32230; Licor) (Figs. 2, 3, Supplementary Fig. S1 panel g). FLAG-Hmx3a proteins were detected with 1:2,000 Monoclonal Anti-FLAG M2 antibody (F1804; Sigma-Aldrich) and either 1:15,000 IRDye 680RD Goat anti-Mouse IgG (925-68070) (Supplementary Fig. S1 panels a-f) or 1:15,000 IRDye 800CW Goat anti-Mouse IgG (925-32210; Licor) (Figs. 2, 3, Supplementary Fig. S1 panel g). Blots were imaged on a Licor Odyssey CLx Imaging System (Licor).

## Supplementary Information

Supplementary Information 1.

Supplementary Information 2.

Supplementary Information 3.

## Data Availability

All original sequencing data from the Y2H screen are available in the NCBI SRA repository under BioProject accession number PRJNA871095 at https://dataview.ncbi.nlm.nih.gov/object/PRJNA871095?reviewer=ajd60kdgt75rl32ipdkd9j16nn.

## References

[1] Wotton KR (2009). Conservation of gene linkage in dispersed vertebrate NK homeobox clusters. Dev. Genes Evol..

[2] Stanfel, M. N., Moses, K. A., Schwartz, R. J. & Zimmer, W. E. Regulation of organ development by the NKX-homeodomain factors: an NKX code. *Cell. Mol. Biol. (Noisy-le-grand).* **Suppl 51**, OL785-99 (2005). 16405855

[3] Wang W, Van De Water T, Lufkin T (1998). Inner ear and maternal reproductive defects in mice lacking the Hmx3 homeobox gene. Development.

[4] Feng Y, Xu Q (2010). Pivotal role of hmx2 and hmx3 in zebrafish inner ear and lateral line development. Dev. Biol..

[5] England SJ (2020). Hmx3a has essential functions in Zebrafish spinal cord, ear and lateral line development. Genetics.

[6] Wang W, Grimmer JF, Van De Water TR, Lufkin T (2004). Hmx2 and Hmx3 homeobox genes direct development of the murine inner ear and hypothalamus and can be functionally replaced by Drosophila Hmx. Dev. Cell.

[7] Hartwell RD (2019). Anteroposterior patterning of the zebrafish ear through Fgf- and Hh-dependent regulation of hmx3a expression. PLOS Genet..

[8] Delile J, Rayon T, Melchionda M, Edwards A, Briscoe J, Sagner A (2019). Single cell transcriptomics reveals spatial and temporal dynamics of gene expression in the developing mouse spinal cord. Development.

[9] Golemis EA (2000). Interaction trap/two-hybrid system to identify interacting proteins. Curr. Protocols Cell Biol..

[10] Smith ST, Jaynes JB (1996). A conserved region of engrailed, shared among all en-, gsc-, Nk1-, Nk2- and msh-class homeoproteins, mediates active transcriptional repression in vivo. Development.

[11] Muhr J, Andersson E, Persson M, Jessell TM, Ericson J (2001). Groucho-mediated transcriptional repression establishes progenitor cell pattern and neuronal fate in the ventral neural tube. Biophysics.

[12] Todd KJ (2012). Establishment of motor neuron-V3 interneuron progenitor domain boundary in ventral spinal cord requires groucho-mediated transcriptional corepression. PLoS ONE.

[13] Grbavec D, Stifani S (1996). Molecular interaction between TLE1 and the carboxyl-terminal domain of HES-1 containing the WRPW motif. Biochem. Biophys. Res. Commun..

[14] Bayramov AV, Martynova NY, Eroshkin FM, Ermakova GV, Zaraisky AG (2004). The homeodomain-containing transcription factor X-nkx-5.1 inhibits expression of the homeobox gene Xanf-1 during the *Xenopus laevis* forebrain development. Mech. Dev..

[15] West KL (2001). HMGN3a and HMGN3b, two protein isoforms with a tissue-specific expression pattern, expand the cellular repertoire of nucleosome-binding proteins. J. Biol. Chem..

[16] Ito Y, Bustin M (2002). Immunohistochemical localization of the nucleosome-binding protein HMGN3 in mouse brain. J. Histochem. Cytochem..

[17] Ueda T, Furusawa T, Kurahashi T, Tessarollo L, Bustin M (2009). The nucleosome binding protein HMGN3 modulates the transcription profile of pancreatic cells and affects insulin secretion. Mol. Cell. Biol..

[18] Thisse C, Thisse B (2008). High-resolution in situ hybridization to whole-mount zebrafish embryos. Nat. Protoc..

[19] Visel A (2004). GenePaint.org: An atlas of gene expression patterns in the mouse embryo. Nucleic Acids Res..

[20] Moleri S (2011). The HMGB protein gene family in zebrafish: Evolution and embryonic expression patterns. Gene Expr. Patterns.

[21] Farnsworth DR, Saunders LM, Miller AC (2020). A single-cell transcriptome atlas for zebrafish development. Dev. Biol..

[22] Tang H (2009). Role of ornithine decarboxylase antizyme inhibitor in vivo. Genes Cells.

[23] Murakami Y, Ichiba T, Matsufuji S, Hayashi SI (1996). Cloning of antizyme inhibitor, a highly homologous protein to ornithine decarboxylase. J. Biol. Chem..

[24] Pegg AE (2006). Regulation of ornithine decarboxylase. J. Biol. Chem..

[25] Pegg AE (2016). Functions of polyamines in mammals. J. Biol. Chem..

[26] Bowie D (2018). Polyamine-mediated channel block of ionotropic glutamate receptors and its regulation by auxiliary proteins. J. Biol. Chem..

[27] Bowie D, Mayer ML (1995). Inward rectification of both AMPA and kainate subtype glutamate receptors generated by polyamine-mediated ion channel block. Neuron.

[28] Aizenman CD, Muñoz-Elías G, Cline HT (2002). Visually driven modulation of glutamatergic synaptic transmission is mediated by the regulation of intracellular polyamines. Neuron.

[29] Root CM, Velázquez-Ulloa NA, Monsalve GC, Minakova E, Spitzer NC (2008). Embryonically expressed GABA and glutamate drive electrical activity regulating neurotransmitter specification. J. Neurosci..

[30] Borodinsky LN (2004). Activity-dependent homeostatic specification of transmitter expression in embryonic neurons. Nature.

[31] Heller JS, Fong WF, Canellakis ES (1976). Induction of a protein inhibitor to ornithine decarboxylase by the end products of its reaction. Proc. Natl. Acad. Sci. U. S. A..

[32] Murakami Y, Hayashi S (1985). Role of antizyme in degradation of ornithine decarboxylase in HTC cells. Biochem. J..

[33] Liu YC, Liu YL, Su JY, Liu GY, Hung HC (2011). Critical factors governing the difference in antizyme-binding affinities between human ornithine decarboxylase and antizyme inhibitor. PLoS One.

[34] Wang F (2021). A comprehensive RNA editome reveals that edited Azin1 partners with DDX1 to enable hematopoietic stem cell differentiation. Blood.

[35] Blythe SA, Cha SW, Tadjuidje E, Heasman J, Klein PS (2010). β-catenin primes organizer gene expression by recruiting a histone H3 arginine 8 methyltransferase, Prmt2. Dev. Cell.

[36] Lakowski TM, Frankel A (2009). Kinetic analysis of human protein arginine N-methyltransferase 2: Formation of monomethyl- and asymmetric dimethyl-arginine residues on histone H4. Biochem. J..

[37] Freese JL, Pino D, Pleasure SJ (2010). Wnt signaling in development and disease. Neurobiol. Dis..

[38] Brantjes H (2001). All Tcf HMG box transcription factors interact with Groucho-related co-repressors. Nucleic Acids Res..

[39] Chen G, Nguyen PH, Courey AJ (1998). A role for Groucho tetramerization in transcriptional repression. Mol. Cell. Biol..

[40] Riz I (2009). Transcriptional activation by TLX1/HOX11 involves Gro/TLE corepressors. Biochem. Biophys. Res. Commun..

[41] Cheng L (2004). Tlx3 and Tlx1 are post-mitotic selector genes determining glutamatergic over GABAergic cell fates. Nat. Neurosci..

[42] Cheng L (2005). Lbx1 and Tlx3 are opposing switches in determining GABAergic versus glutamatergic transmitter phenotypes. Nat Neurosci.

[43] Lu FI, Sun YH, Wei CY, Thisse C, Thisse B (2014). Tissue-specific derepression of TCF/LEF controls the activity of the Wnt/β-catenin pathway. Nat. Commun..

[44] Dintilhac A, Bernués J (2002). HMGB1 interacts with many apparently unrelated proteins by recognizing short amino acid sequences. J. Biol. Chem..

[45] Paroush Z (1994). Groucho is required for Drosophila neurogenesis, segmentation, and sex determination and interacts directly with hairy-related bHLH proteins. Cell.

[46] Fisher AL, Ohsako S, Caudy M (1996). The WRPW motif of the hairy-related basic helix-loop-helix repressor proteins acts as a 4-amino-acid transcription repression and protein-protein interaction domain. Mol. Cell. Biol..

[47] Reeves R (2015). High mobility group (HMG) proteins: Modulators of chromatin structure and DNA repair in mammalian cells. DNA Repair.

[48] Ueda T, Yoshida M (2010). HMGB proteins and transcriptional regulation. Biochim. Biophys. Acta - Gene Regul. Mech..

[49] Zhu N, Hansen U (2010). Transcriptional regulation by HMGN proteins. Biochim. Biophys. Acta.

[50] Furusawa T, Cherukuri S (2010). Developmental function of HMGN proteins. Biochim. Biophys. Acta - Gene Regul. Mech..

[51] Taniguchi N (2009). Chromatin protein HMGB2 regulates articular cartilage surface maintenance via β-catenin pathway. Proc. Natl. Acad. Sci. U. S. A..

[52] Zhao X (2011). High mobility group box-1 (HMGB1; amphoterin) is required for zebrafish brain development. J. Biol. Chem..

[53] Itou J (2011). HMGB factors are required for posterior digit development through integrating signaling pathway activities. Dev. Dyn..

[54] Dorsky RI, Vincan E (2009). Neural patterning and CNS functions of Wnt in zebrafish. Wnt Signaling.

[55] Riccomagno MM, Takada S, Epstein DJ (2005). Wnt-dependent regulation of inner ear morphogenesis is balanced by the opposing and supporting roles of Shh. Genes Dev..

[56] Bajoghli B, Aghaallaei N, Jung G, Czerny T (2009). Induction of otic structures by canonical Wnt signalling in medaka. Dev. Genes Evol..

[57] Aman A, Nguyen M, Piotrowski T (2011). Wnt/β-catenin dependent cell proliferation underlies segmented lateral line morphogenesis. Dev. Biol..

[58] Dorsky RI, Sheldahl LC, Moon RT (2002). A transgenic lef1/β-catenin-dependent reporter is expressed in spatially restricted domains throughout zebrafish development. Dev. Biol..

[59] Trapnell C (2012). Differential gene and transcript expression analysis of RNA-seq experiments with TopHat and Cufflinks. Nat. Protoc..

[60] Robinson JT (2011). Integrative genome viewer. Nat. Biotechnol..

[61] Ruzicka L (2019). The Zebrafish Information Network: new support for non-coding genes, richer Gene Ontology annotations and the Alliance of Genome Resources. Nucleic Acids Res..

[62] Howe KL (2021). Ensembl 2021. Nucleic Acids Res..

[63] UniProt: the universal protein knowledgebase in 2021. *Nucleic Acids Res.* **49**, D480–D489 (2021). 10.1093/nar/gkaa1100 PMC7778908 33237286

