# Supplementary material for: Analyses of binding partners and functional domains for the developmentally essential protein Hmx3a/HMX3
Source: Sci Rep. 2023 Jan 20;13:1151. doi: 10.1038/s41598-023-27878-9 (PMC9859826; doi:10.1038/s41598-023-27878-9)
Supplement: Supplementary file 1 — Supplementary Information 1. [file 41598_2023_27878_MOESM1_ESM.pdf]

Supplementary Information for:

Analyses of binding partners and functional domains for the developmentally essential protein Hmx3a/HMX3

William Haws[1], Samantha England[1], Ginny Grieb[1], Gabriela Susana[1], Sophie Hernandez[1], Hunter Mirer[1], and Katharine Lewis[1*]

[1]Department of Biology, Syracuse University, New York 13244

[*]Corresponding author. Department of Biology, Syracuse University, 107 College Place, Syracuse, NY 13244, USA. Telephone: +1 315 443 5902; Email: kelewi02@syr.edu

Supplementary Figure S1: Zebrafish Rack1, Sdcbp, Wdr61, Fez1, Calr, and Oaz1a do not bind full-length zebrafish Hmx3a in Co-IPs

Putative zebrafish binding partners were expressed as GST-fusion proteins, purified, and incubated with either a FLAG-Hmx3a-expressing embryo lysate or a stage-matched control lysate expressing no recombinant Hmx3a. Proteins were immunoprecipitated with an anti-FLAG antibody and separated by SDS-PAGE followed by transfer to a membrane and immunoblotting with anti-FLAG or anti-GST antibody. Top images in each panel show anti-GST immunoblot (IB) of 4% of input. Middle and bottom images in each panel show blots for GST and FLAG, respectively, of the immunoprecipitate (IP). The Hmx3a protein included in each lysate is indicated along the top *x* axis. MW is shown on the right-hand side. Prey proteins were **(a)** Hmgb1a (included as a positive control with these antibody conditions – see figure 2 and results), **(b)** Rack1, **(c)** Sdcbp, **(d)** Wdr61, **(e)** Fez1, **(f)** Calr, and **(g)** Oaz1a. Red arrowhead in **(f)** indicates full-length GST-Calr band; most prominent band in all other input fractions corresponds to full-length prey protein. FLAG antibody heavy chain appears at just above 50 kDa in IP:FLAG;IB:FLAG fractions. Non-specific signal from heavy chain is also slightly visible in IP:FLAG;IB:GST fractions (bands just above 50 kDa of equal intensity between control and +Hmx3a lanes). n = at least 2 for all experiments. Original blots are presented in Supplementary Fig. S3.

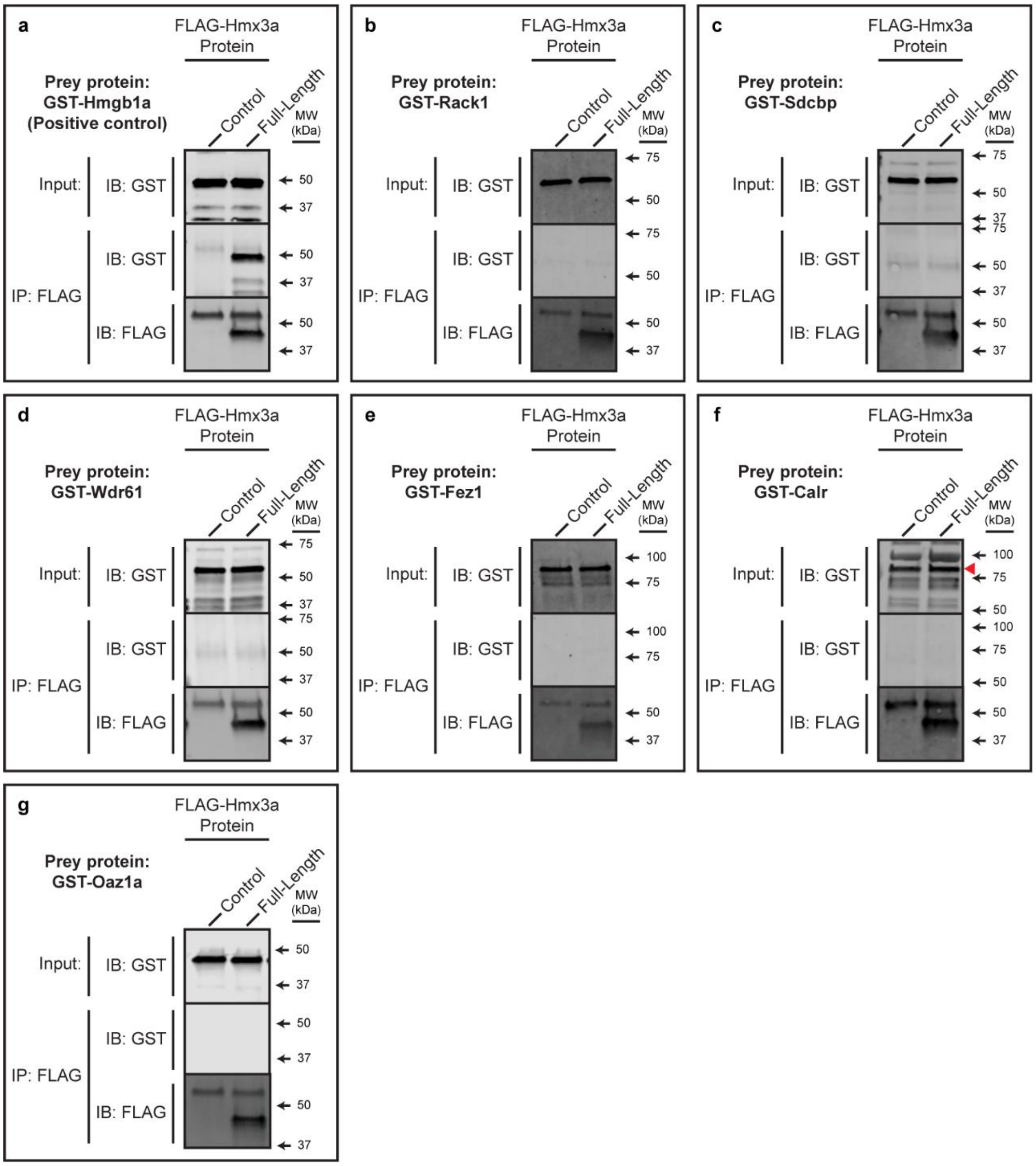

a
FLAG-Hmx3a Protein
Prey protein: GST-Hmgb1a (Positive control)
Control
Full-Length
MW (kDa)
Input:
IB: GST
IP: FLAG
IB: GST
IB: FLAG
50
37
b
Prey protein: GST-Rack1
75
c
Prey protein: GST-Sdcbp
d
Prey protein: GST-Wdr61
e
Prey protein: GST-Fez1
100
f
Prey protein: GST-Calr
g
Prey protein: GST-Oaz1a

```
Zebrafish_Hmx3a    1 MPETTQDTCASA--------------KDSPFFIKNLLNSD--SKPSKPKP     34
                     |||...|...:|              |:|||.|:||||.|  ..|.||:|
Mouse_HMX3         1 MPEPGPDASGTASAPPPQPPPQPPAPKESPFSIRNLLNGDHHRPPPKPQP     50

Zebrafish_Hmx3a   35 ----ILAP--------------TKAGLDGS--FSLSQVGEINFPRFELPT     64
                         :.||              .|..|:|:  |:|||||::.|||||:|.
Mouse_HMX3        51 PPRTLFAPASAAAAAAAAAAAAAKGALEGAAGFALSQVGDLAFPRFEIPA    100

Zebrafish_Hmx3a   65 QRFALPA-YLERASAWWYPYTLS-ASAHLHRTEAAQKA--RDSSPTTGTD    110
                     ||||||| ||||:.||||||||: |..||.|.||::||  |||||.:|||
Mouse_HMX3       101 QRFALPAHYLERSPAWWYPYTLTPAGGHLPRPEASEKALLRDSSPASGTD    150

Zebrafish_Hmx3a  111 RDSPELVLKSDPDAKDDEDDNKSGDEIVLEESDTEDGKKEG---------    151
                     |||||.:||:|||.|  |.|:||.|||:|||||:|:|||||
Mouse_HMX3       151 RDSPEPLLKADPDHK--ELDSKSPDEIILEESDSEEGKKEGEAVPGAAGT    198

Zebrafish_Hmx3a  152 ---------GIDDWKKSDDGADKKP-CRKKKTRTVFSRSQVFQLESTFDM    191
                              |.:|||...:..:||| ||||||||||||||||||||||||
Mouse_HMX3       199 TVGATTATPGSEDWKAGAESPEKKPACRKKKTRTVFSRSQVFQLESTFDM    248

Zebrafish_Hmx3a  192 KRYLSSSERAGLAASLHLTETQVKIWFQNRRNKWKRQLAAELEAANLSHA    241
                     ||||||||||||||||||||||||||||||||||||||||||||||||||
Mouse_HMX3       249 KRYLSSSERAGLAASLHLTETQVKIWFQNRRNKWKRQLAAELEAANLSHA    298

Zebrafish_Hmx3a  242 AAQRIVRVPILYHENSASESTNTAGN--VPVSQPLLTFPHPVYYSHPIVT    289
                     |||||||||||||||||:|....|..  |||||||||||||||||||:|:
Mouse_HMX3       299 AAQRIVRVPILYHENSAAEGAAAAAGAPVPVSQPLLTFPHPVYYSHPVVS    348

Zebrafish_Hmx3a  290 SVPLLRPV    297
                     ||||||||
Mouse_HMX3       349 SVPLLRPV    356
```

Supplemental Figure S2: Zebrafish Hmx3a and Mouse HMX3 Annotated Alignment

Zebrafish Hmx3a and Mouse HMX3 were aligned using EMBOSS Needle (Madeira et al. (2022) Search and sequence analysis tools services from EMBL-EBI in 2022. *Nucleic Acids Research* **50**, W276). A single dot indicates dissimilar residues and two dots (:) indicate similar residues. Yellow indicates the eh1A domain; green indicates the eh1B domain and blue indicates the WYPY domain.

Supplementary Figure S3: Original blots from indicated figures and panels.
Cropped images bounded in black boxes are replicated from the indicated figure/panel and were cropped from the full-membrane black and white images. Red dashed box indicates section of image cropped and inserted into associated figure and panel. In cases where molecular weight marker bands and/or membrane edges don't show in black and white image, an additional two-color image is provided. In two-color blots, green is the 800 nm imaging channel and red is the 700 nm channel (see antibody information in methods section). In blots from Fig. 2, Fig. 3, and Supplementary Fig. S1 panel g, both GST and FLAG appear in the 800 nm channel. In other blots from Supplementary Fig. S1, anti-GST and associated secondary antibody signal appears in the 800 nm channel and anti-FLAG and associated secondary antibody in the 700 nm channel. Molecular weight marker bands are, from largest (top) to smallest (bottom): 250, 150, 100, 75, 50, 37, 25, 20, 15, and 10 kDa. All molecular weight marker bands are visible in the 700 nm channel. 75 kDa and 25 kDa reference bands are always visible in both channels.

Figure 2, Panel b

Input; IB: GST

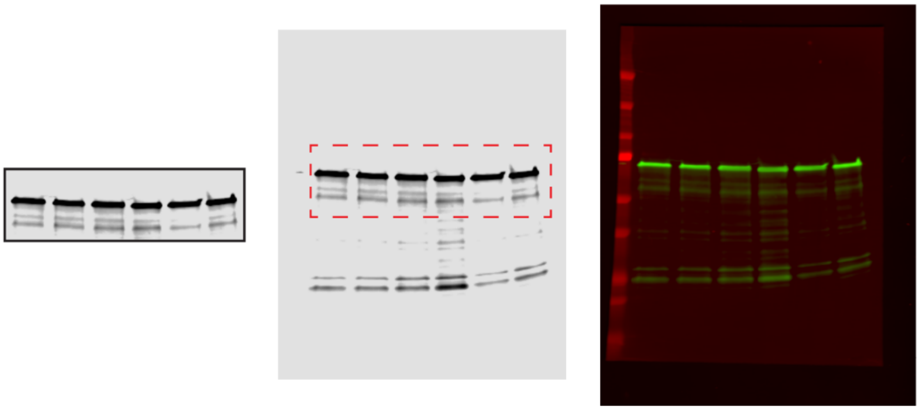

IP: FLAG; IB: GST

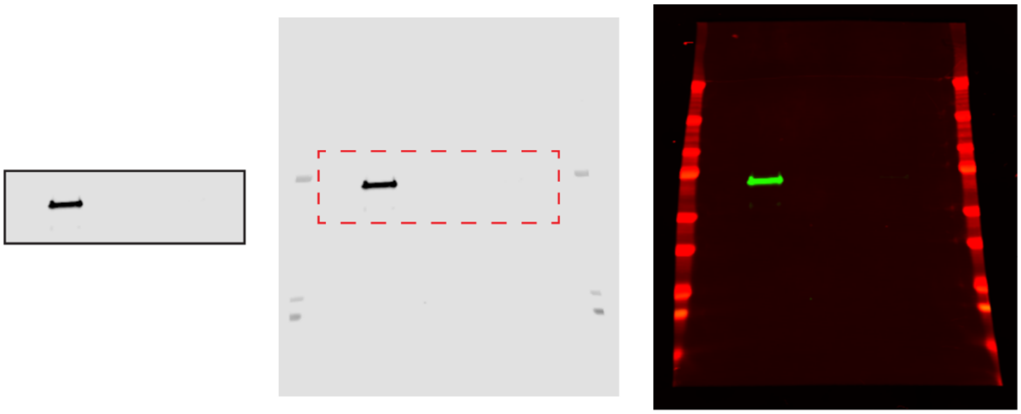

IP: FLAG IB: FLAG

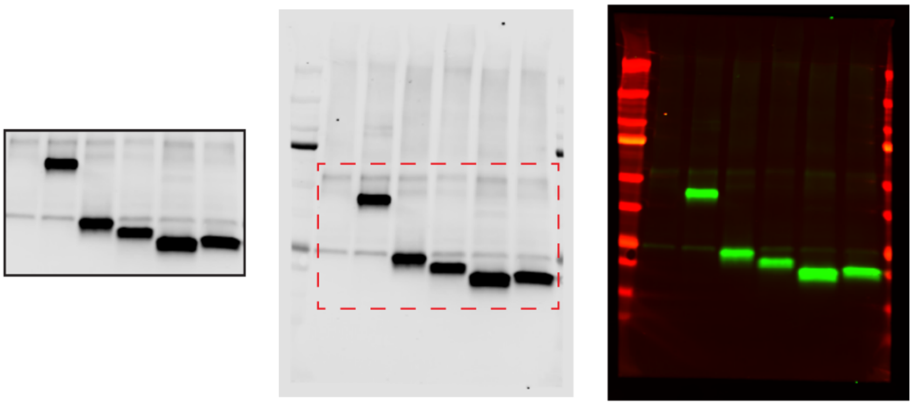

Figure 2, Panel c

Input; IB: GST

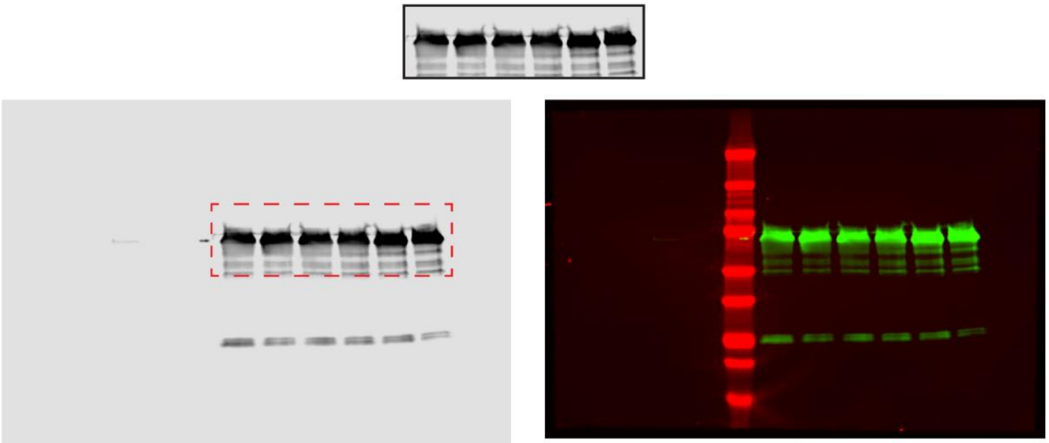

IP: FLAG; IB: GST

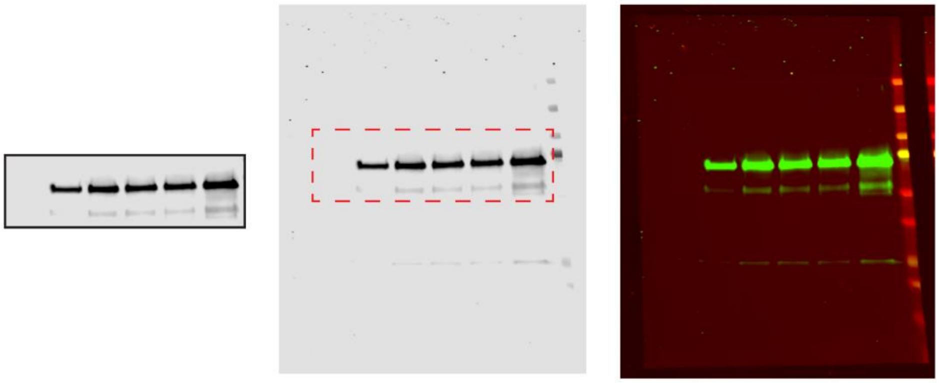

IP: FLAG IB: FLAG

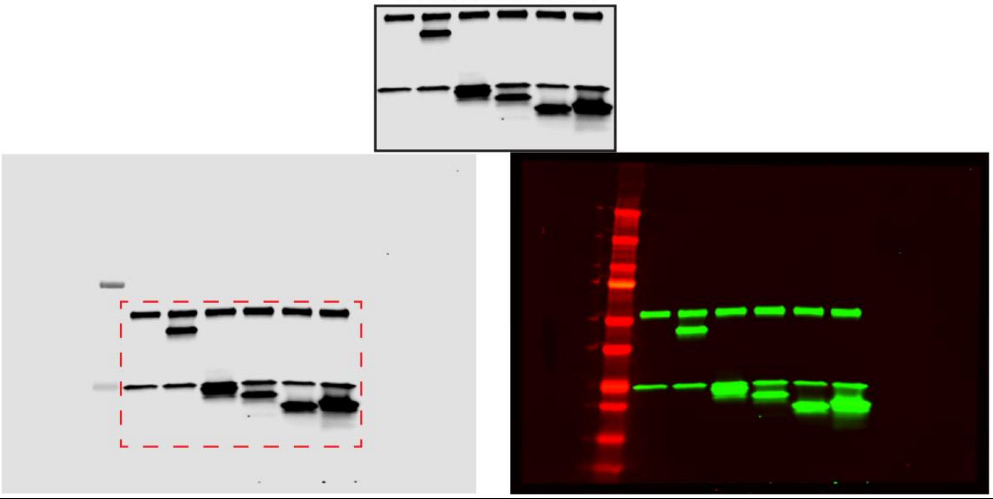

Figure 2, Panel d

Input; IB: GST

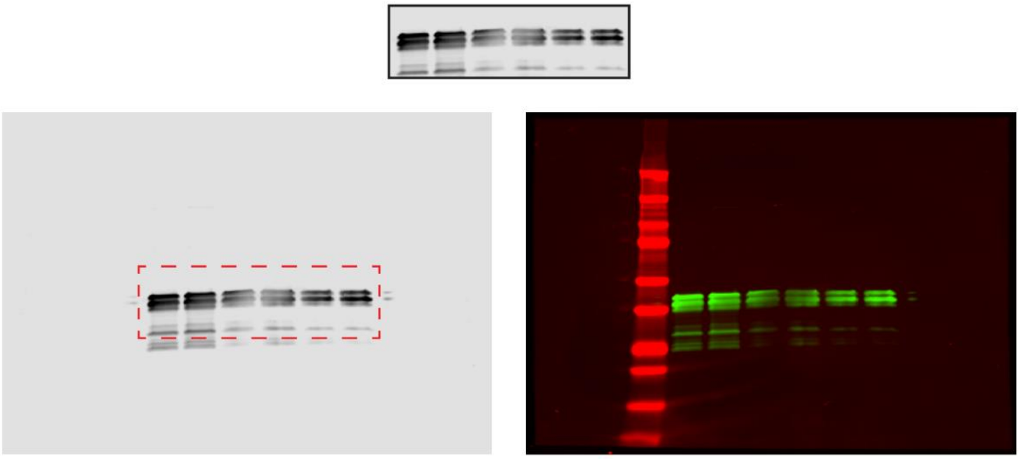

IP: FLAG; IB: GST

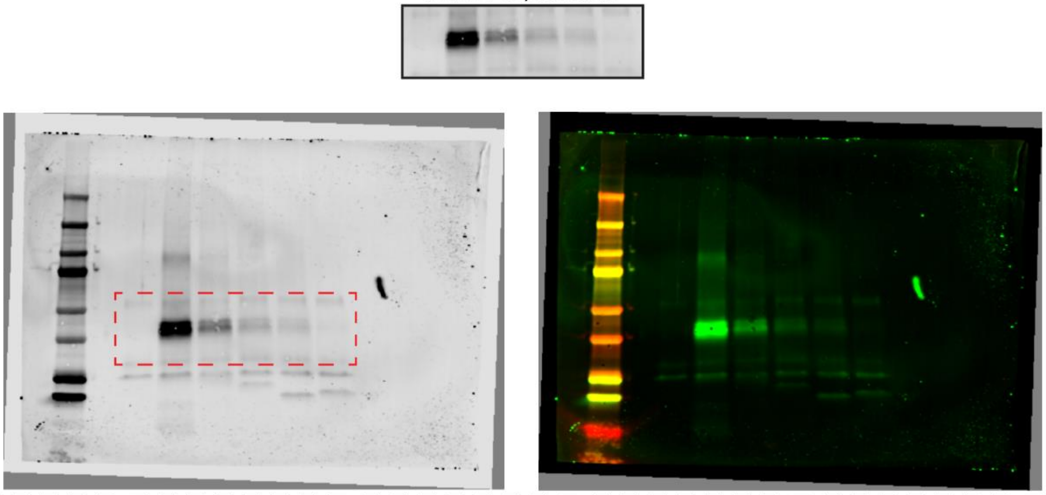

IP: FLAG IB: FLAG

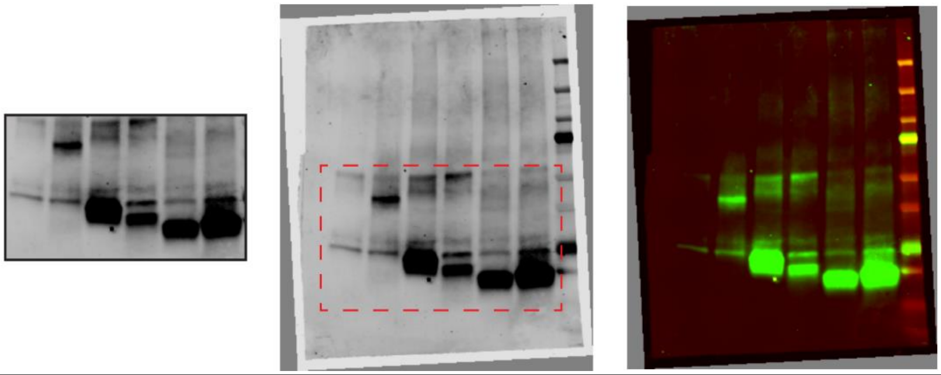

Figure 2, Panel e

Input; IB: GST

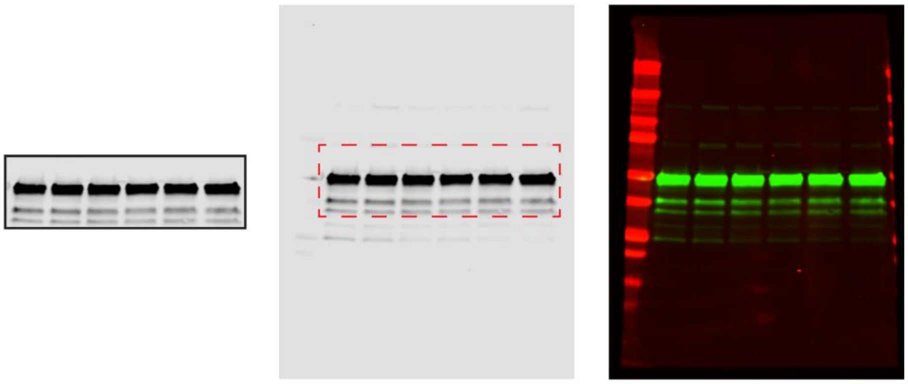

IP: FLAG; IB: GST

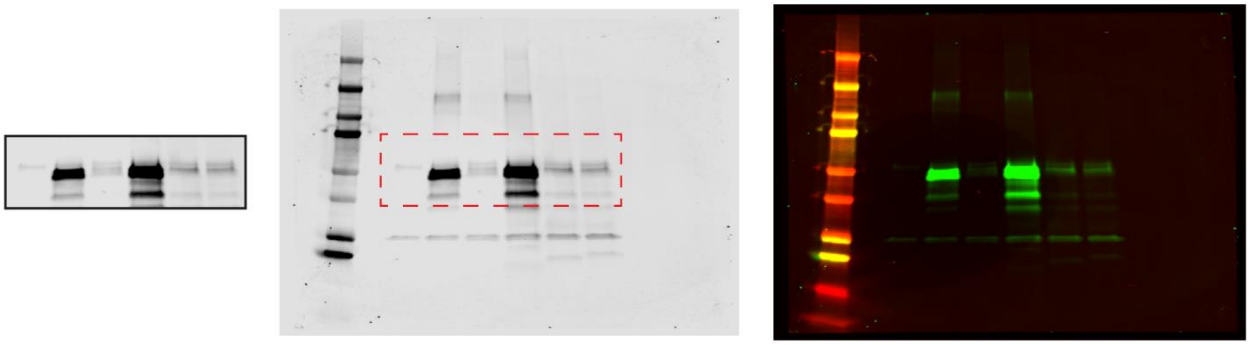

IP: FLAG IB: FLAG

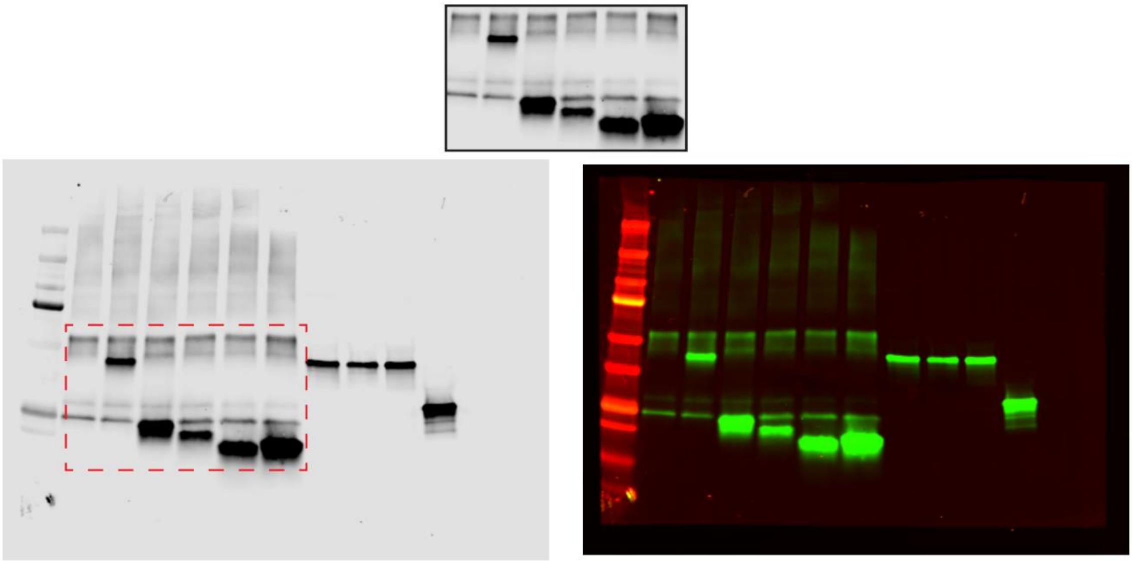

Figure 3, Panel b

Input; IB: GST

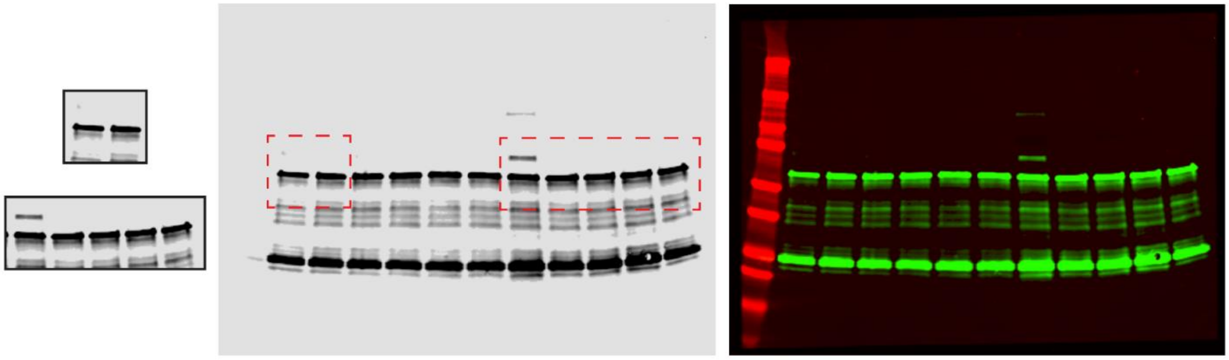

IP: FLAG; IB: GST

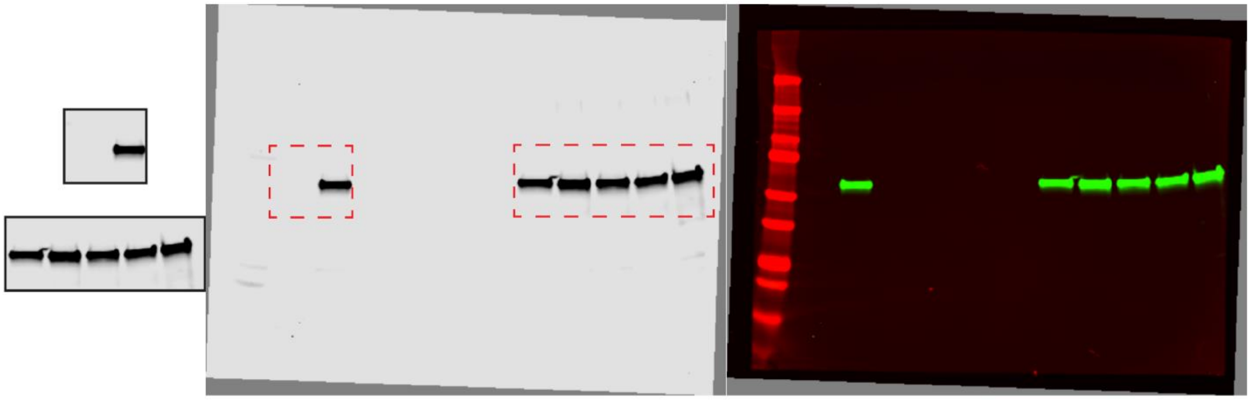

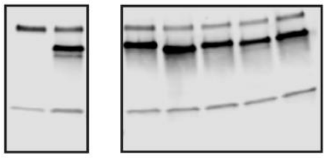

IP: FLAG IB: FLAG

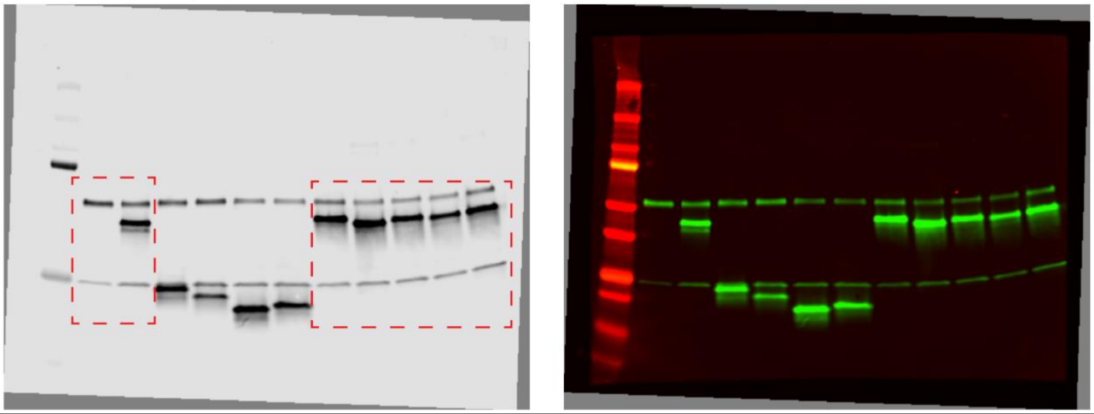

Figure 3, Panel c

Input; IB: GST

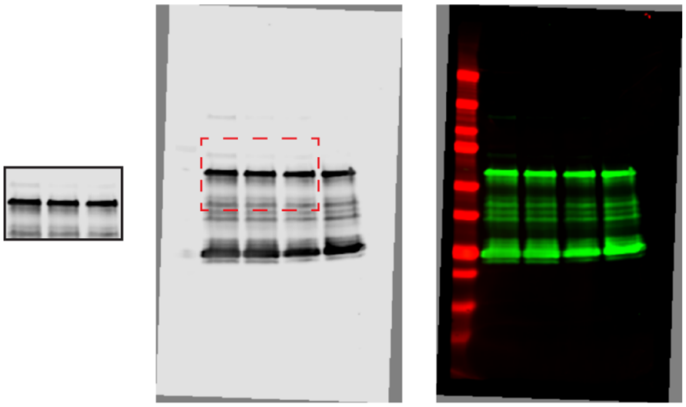

IP: FLAG; IB: GST

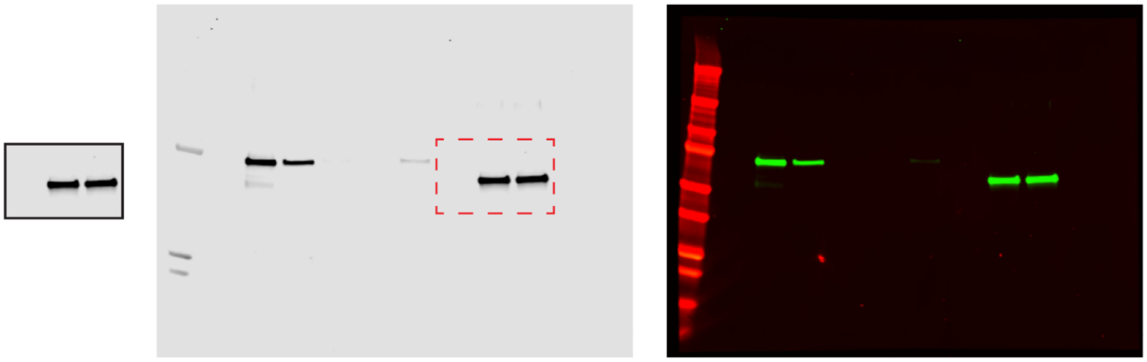

IP: FLAG IB: FLAG

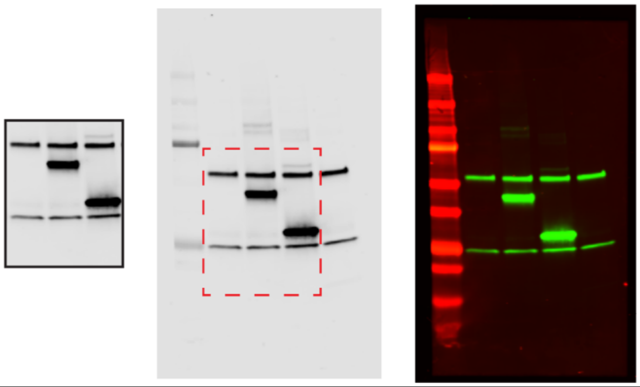

Supplementary Figure S1, Panel a

Input; IB: GST

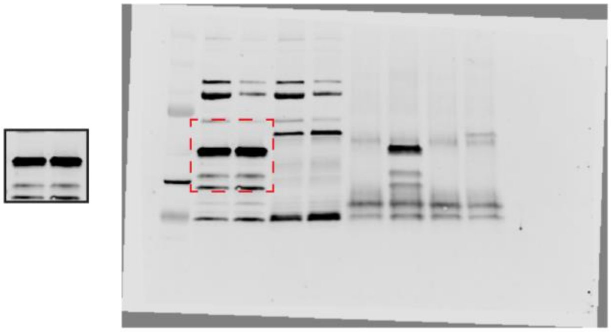

IP: FLAG; IB: GST

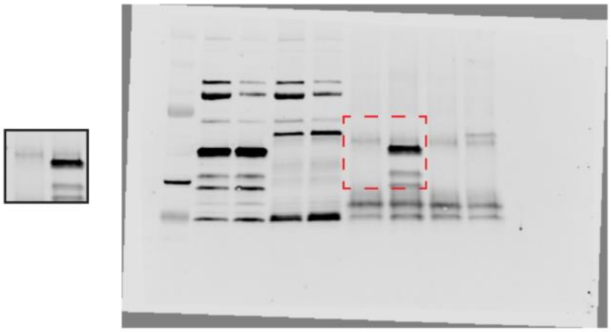

IP: FLAG IB: FLAG

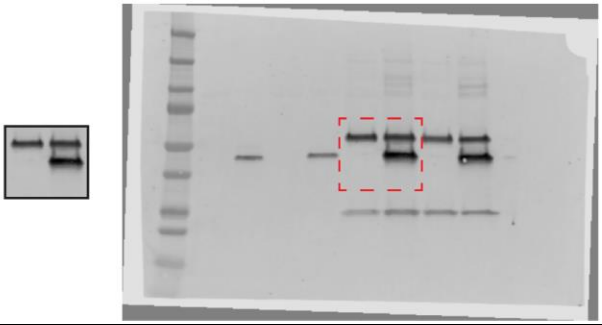

Supplementary Figure S1, Panel b

Input; IB: GST

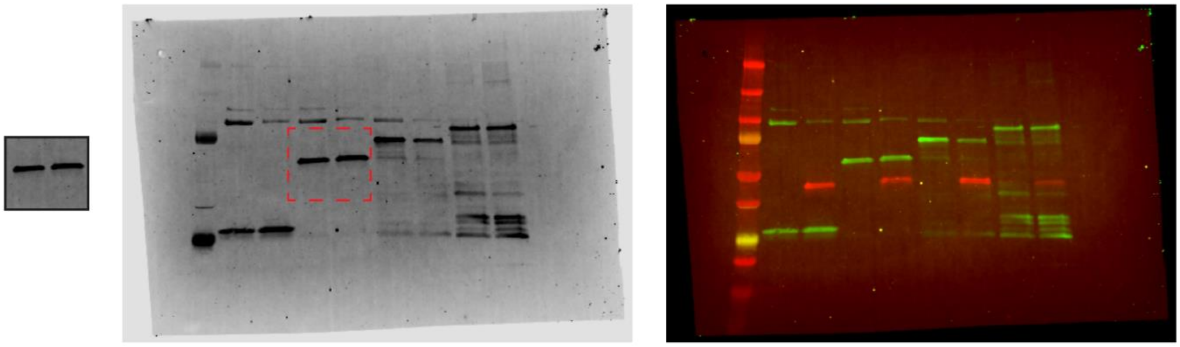

IP: FLAG; IB: GST

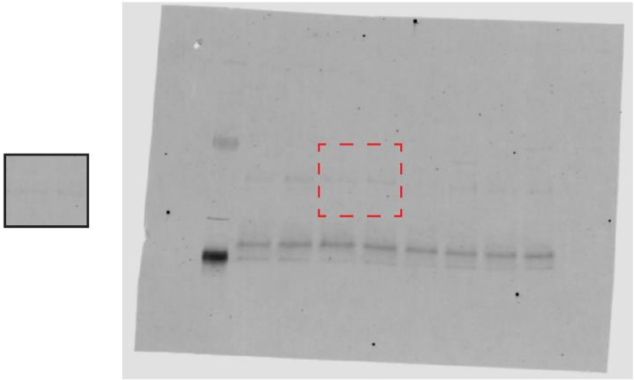

IP: FLAG IB: FLAG

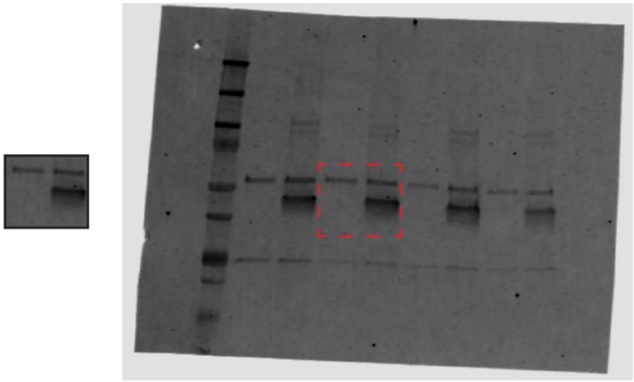

Supplementary Figure S1, Panel c

Input; IB: GST

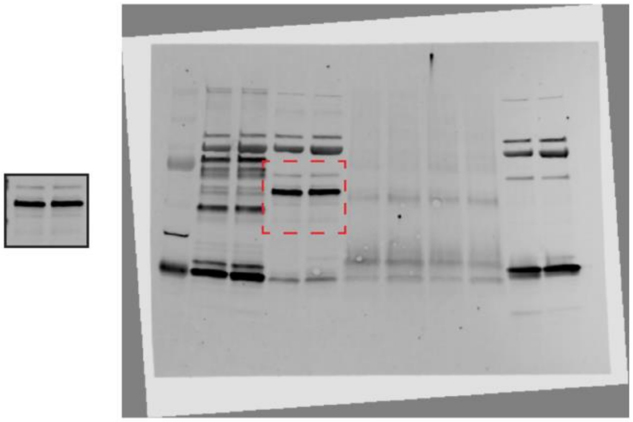

IP: FLAG; IB: GST

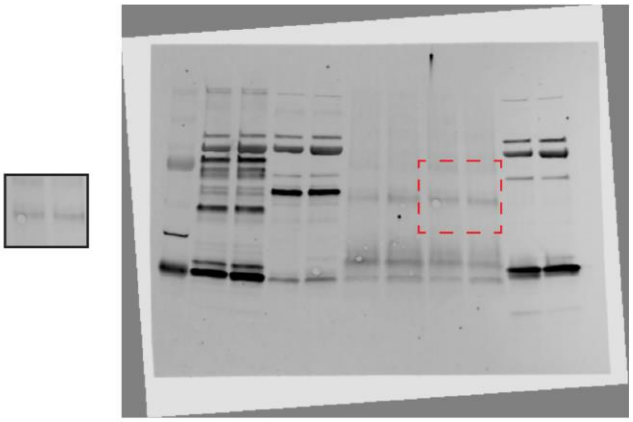

IP: FLAG IB: FLAG

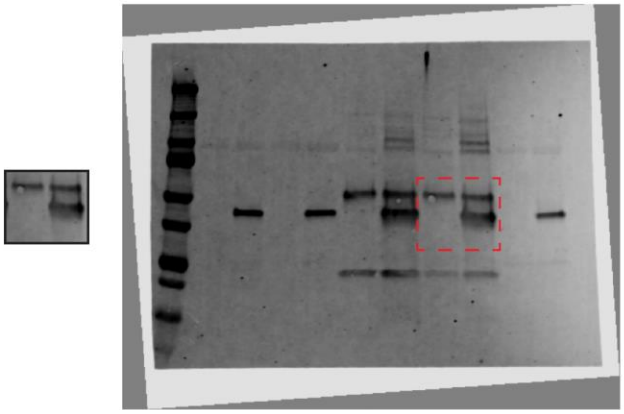

Supplementary Figure S1, Panel d

Input; IB: GST

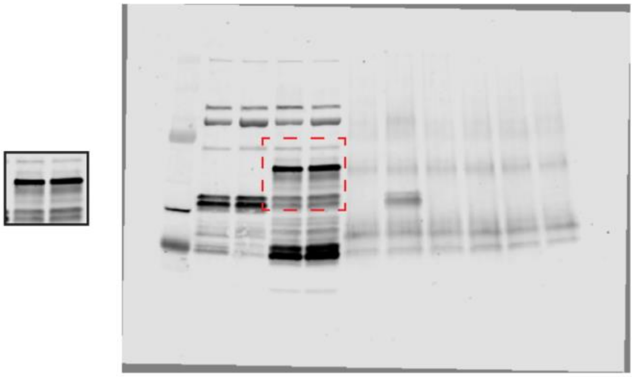

IP: FLAG; IB: GST

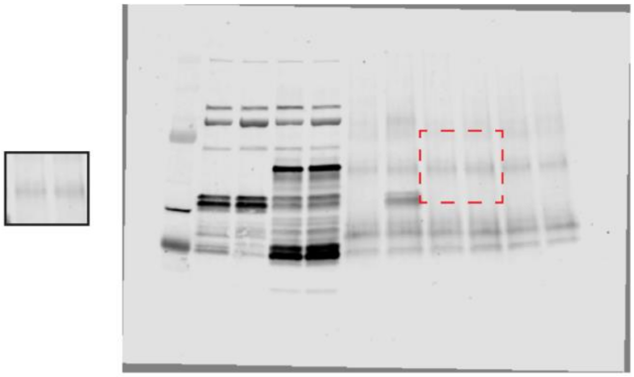

IP: FLAG IB: FLAG

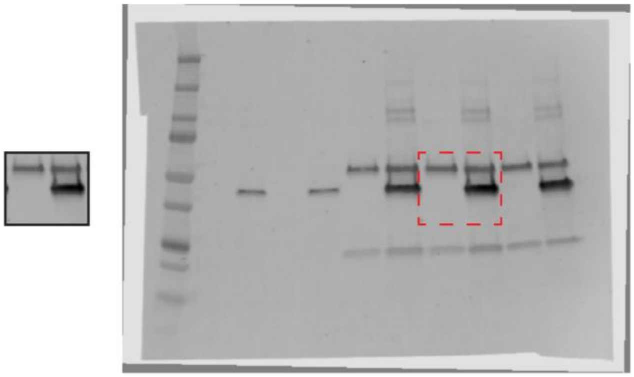

Supplementary Figure S1, Panel e

Input; IB: GST

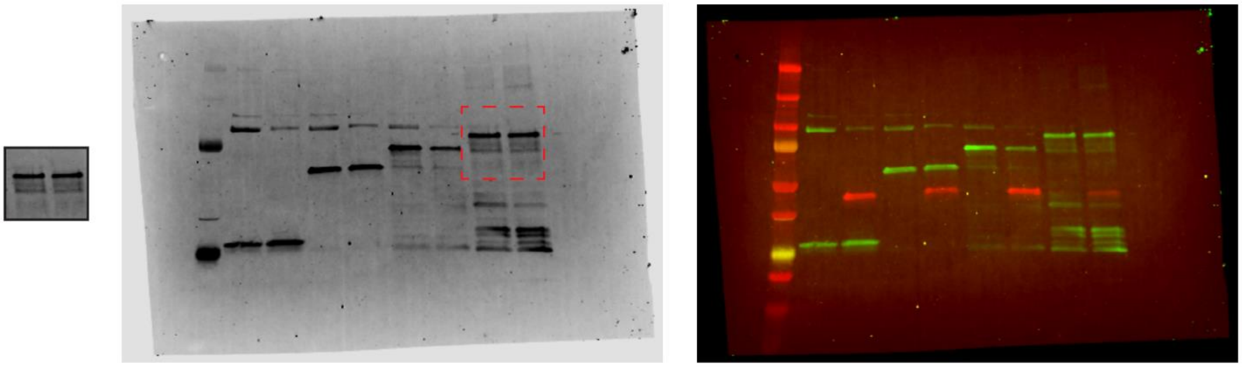

IP: FLAG; IB: GST

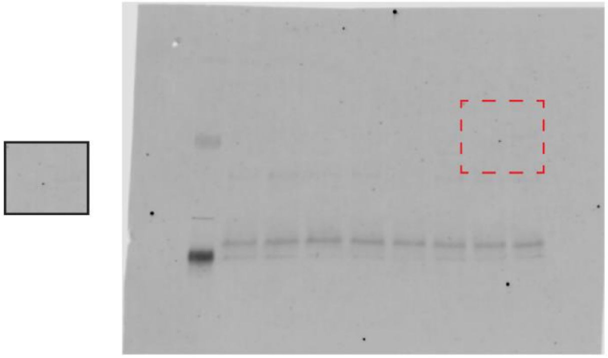

IP: FLAG IB: FLAG

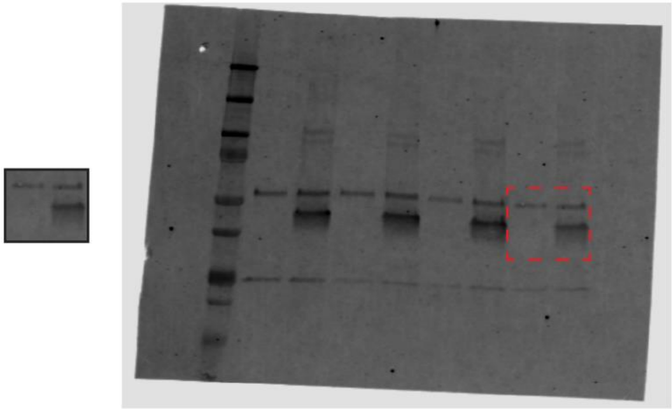

Supplementary Figure S1, Panel f

Input; IB: GST

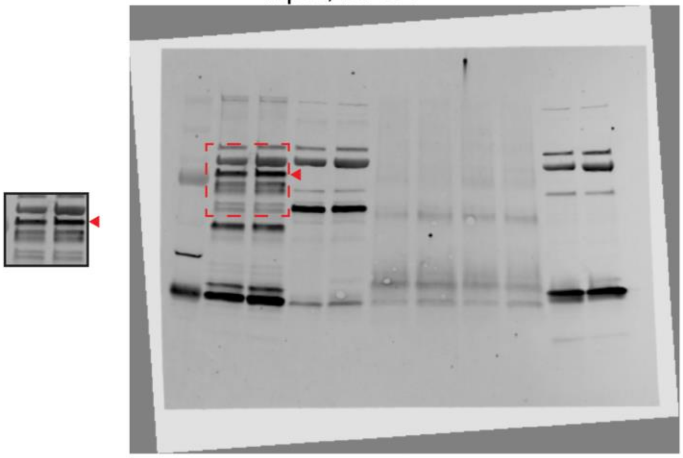

IP: FLAG; IB: GST

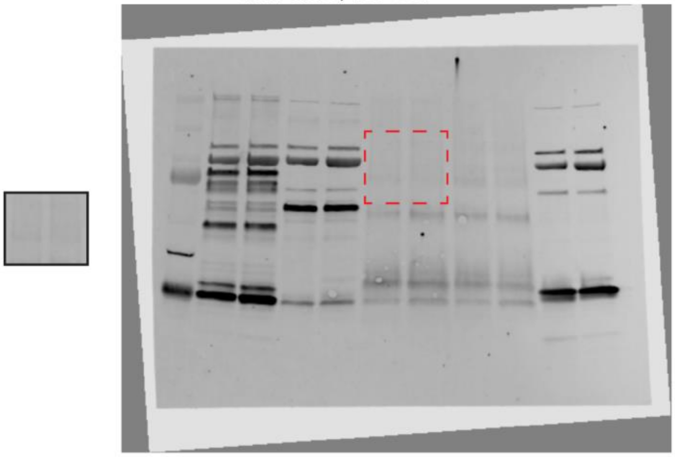

IP: FLAG IB: FLAG

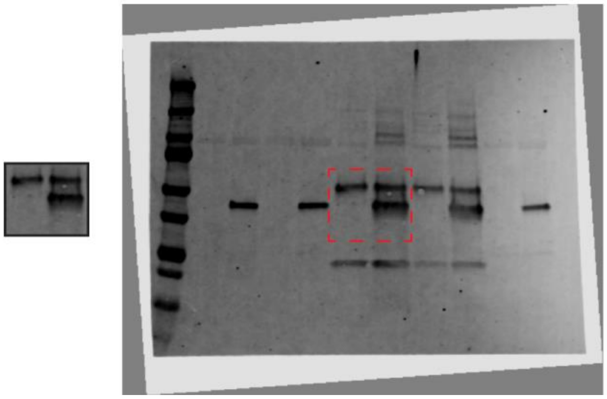

Supplementary Figure S1, Panel g

Input; IB: GST

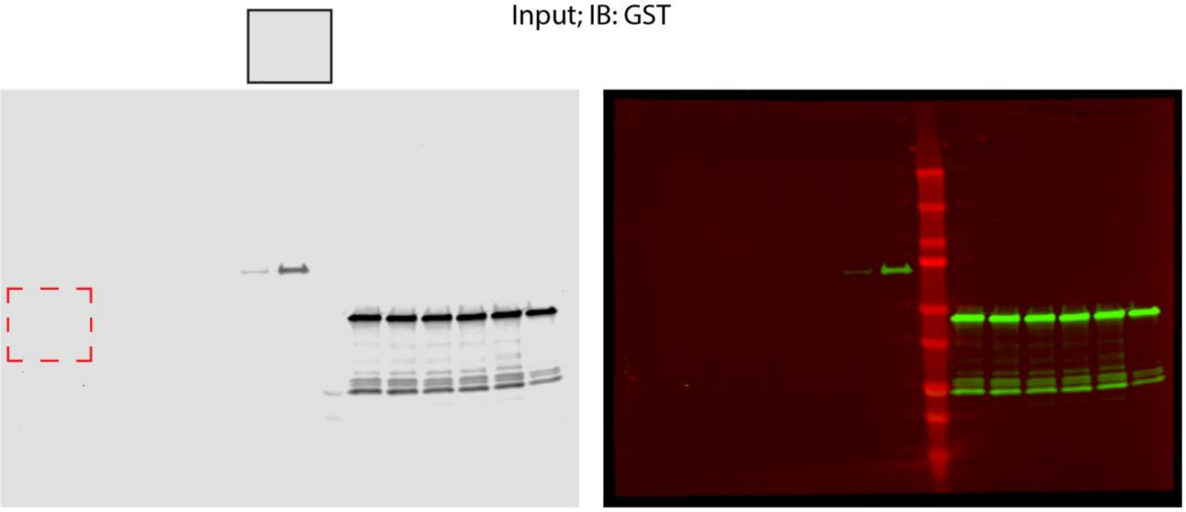

IP: FLAG; IB: GST

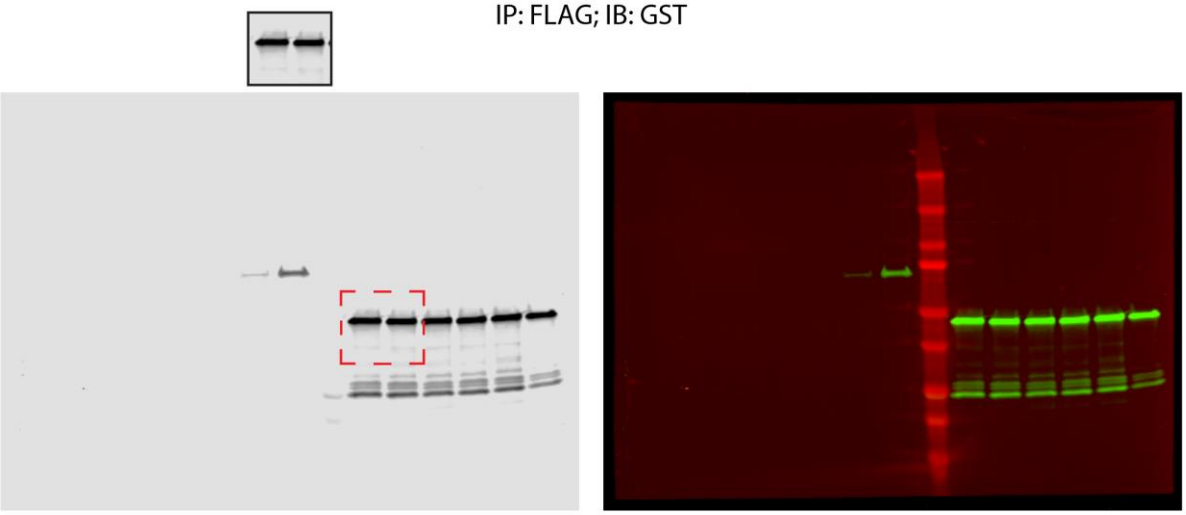

IP: FLAG IB: FLAG

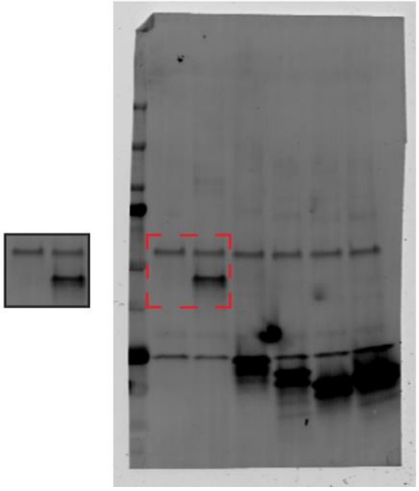

Supplementary Table Legends

Supplementary Table S1: Full list of HMX3 binding partners identified with yeast two-hybrid screen. Table lists all of the mouse genes identified by sequencing of positive clones from the yeast two-hybrid screen. Column 1 provides the gene symbol, column 2 the number of reads that aligned to that gene, column 3 the MGI Accession IDs for each gene, and column 4 the full gene name. * indicates genes for which zebrafish orthologs were confirmed to bind zebrafish Hmx3a using Co-IPs. ** indicates genes for which zebrafish orthologs did not bind zebrafish Hmx3a in Co-IPs. *** indicates protein-coding genes with a pseudogene annotated at the same locus, which were not assigned a read count in automated analysis.

Supplementary Table S2: Primers and PCR conditions used in this study. Table shows the primers and PCR conditions for all of the PCR amplifications conducted in this study. * indicates primers used for Sanger sequencing of respective constructs. ** *pCS2-FLAG-Hmx3a-∆WYPY* was sequenced with a primer of sequence 5’ CTTTGAGTTACCCACCCAGC 3’. *** *pCS2-FLAG-Hmx3a-∆eh1A∆eh1B∆WYPY* was sequenced with a primer of sequence 5’ TCTTCCAGTCGTCTATGCCG 3’.
